# Retro-inverso Urokinase Receptor Antagonists for the Treatment of Metastatic Sarcomas

**DOI:** 10.1038/s41598-017-01425-9

**Published:** 2017-05-02

**Authors:** Maria Vincenza Carriero, Katia Bifulco, Vincenzo Ingangi, Susan Costantini, Giovanni Botti, Concetta Ragone, Michele Minopoli, Maria Letizia Motti, Domenica Rea, Giosuè Scognamiglio, Gerardo Botti, Claudio Arra, Gennaro Ciliberto, Antonello Pessi

**Affiliations:** 1 IRCCS Istituto Nazionale Tumori “Fondazione G. Pascale”, Naples, Italy; 2 Peptipharma, Viale Città D’Europa 679, 00144 Rome, Italy; 3 Scientific Directorate IRCCS Istituto Nazionale Tumori Regina Elena, Rome, Italy

## Abstract

The development of metastases is a multistep process that requires the activation of physiological and biochemical processes that govern migration, invasion and entry of metastatic cells into blood vessels. The urokinase receptor (uPAR) promotes cell migration by interacting with the Formyl Peptide Receptors (FPRs). Since both uPAR and FPR1 are involved in tumor progression, the uPAR-FPR1 interaction is an attractive therapeutic target. We previously described peptide antagonists of the uPAR-FPR1 interaction that inhibited cell migration and angiogenesis. To develop enzyme-resistant analogues, we applied here the Retro-Inverso (RI) approach, whereby the topology of the side chains is maintained by inverting the sequence of the peptide and the chirality of all residues. Molecular dynamics suggests that peptide RI-3 adopts the turn structure typical of uPAR-FPR1 antagonists. Accordingly, RI-3 is a nanomolar competitor of N-formyl-Met-Leu-Phe for binding to FPR1 and inhibits migration, invasion, trans-endothelial migration of sarcoma cells and VEGF-triggered endothelial tube formation. When sarcoma cells were subcutaneously injected in nude mice, tumor size, intra-tumoral microvessel density, circulating tumor cells and pulmonary metastases were significantly reduced in animals treated daily with 6 mg/Kg RI-3 as compared to animals treated with vehicle only. Thus, RI-3 represents a promising lead for anti-metastatic drugs.

## Introduction

Despite significant progress in therapy, patients affected by solid tumors frequently die for systemic spread of the disease to distant sites. The development of metastases is a multistep process involving migration from the primary tumor site, invasion through the basement membrane, entry of metastatic cells into the blood vessels and finally, localization to the second site^1^. At the heart of this process is cell migration, a spatially and temporally coordinated process that orchestrates physiological processes such as embryonic morphogenesis, tissue repair and regeneration, and immune-cell trafficking^2^. When cell migration is deregulated, it contributes to numerous disorders including tumor metastasis, chronic inflammation, and vascular disease^3, 4^. Therefore, the control of cell motility is an attractive approach for the clinical management of metastases from solid tumors, including sarcomas, which have high propensity for metastasis to lungs.

The Urinary Plasminogen Activator Receptor (uPAR), also called urokinase receptor, is a widely recognized master regulator of cell migration^5^. uPAR is a glycosylated glycosyl-phosphatidyl-inositol-(GPI)anchored protein^6^, formed by 3 domains (DI-DIII). When expressed on cell surface, uPAR promotes cell-associated proteolysis by binding to Urokinase Plasminogen Activator (uPA), which locally converts plasminogen into active plasmin, thus favoring tissue invasion and metastasis^7, 8^. Plasmin generated by uPA or uPA itself can cleave intact uPAR (DI-DIII), releasing DI, while the remaining GPI-anchored DII‒DIII can remain on cell surface or be secreted in the extracellular milieu following cleavage of the anchor^9^. Full-length uPAR or fragments deriving from its cleavage on the cell surface may be released in soluble form in plasma and/or urine^10^.

The clinical relevance of uPAR as a prognostic marker in human cancers is well documented, and high levels of soluble uPAR in serum are associated with poor prognosis and increased risk of metastasis^10^. Besides being responsible for focusing urokinase-mediated plasminogen activation on cell surface^11^, uPAR also promotes intracellular signaling, this way regulating physiologic processes such as wound healing, immune responses, and stem cell mobilization, as well as pathologic conditions such as inflammation and tumor progression^5, 7^.

We and others have shown that uPAR signaling occurs through the assembly in composite regulatory units with extracellular matrix (ECM) proteins such as vitronectin, with the G protein-coupled Formyl-Peptide Receptors (FPRs), and with integrins^12–19^. Due to the pleiotropic nature of its interactors, uPAR represents both a challenge and an opportunity for drug discovery. However, despite significant effort, no uPAR-targeted therapeutics are in clinical evaluation to date. This supports the relevance of innovative, therapeutic approaches devoted to interfering with uPAR/co-receptor interactions.

The uPAR domains DI-DIII are connected by short linker regions^20^. DI-DIII pack together into a concave structure that shifts to an active conformation upon binding to uPA^21, 22^. The linker between DI-DII is more flexible than that between the DII‒DIII domains^23–25^, and includes the protease-sensitive crucial signaling region, uPAR_84–95_. In the form of a synthetic peptide, the minimal 88–92 sequence (Ser^88^-Arg-Ser-Arg-Tyr^92^, SRSRY) retains chemotactic activity and triggers directional cell migration and angiogenesis *in vitro* and *in vivo* ^15–17, 26, 27^. Mechanistically, these activities are mediated through interaction with the FPR type 1 (FPR1) which, in turn, activates the vitronectin receptor with an inside-out type of mechanism which involves PKC and ERK phosphorylation^17, 27^. Recently, we found that cyclized SRSRY had an opposite effect on cell migration, as compared to its linear form, and inhibited fMLF-induced monocyte locomotion with an IC_50_ value of 10 pM as well as neovascularization and intravasation of osteosarcoma cells^28, 29^. FPRs are a family of 7 transmembrane domain, G-protein-coupled receptors that exert multiple functions in many pathophysiologic processes because of their capacity to interact with a variety of structurally diverse ligands^30, 31^. Human FPR1, originally identified in neutrophils, monocytes and macrophages, elicits many responses upon ligation of formyl-peptide ligands derived from bacteria and/or mitochondria of eukaryotic cells, including morphological polarization, locomotion, production of reactive-oxygen species and release of cytokines and proteolytic enzymes^32^. In recent years, FPR1 has been shown to be expressed also in several non-myeloid cells, and accumulating evidence demonstrates that FPR1 is also involved in progression of solid tumors^32–35^. Therefore, the inhibition of the uPAR/FPR1 interaction represents an attractive target to inhibit the metastatic process in solid tumors.

We previously showed that the substitution of Ser90 with Glu in the chemotactic sequence uPAR_84–95_ prevents agonist-triggered FPR1 activation and internalization^36^. Following on this observation, we developed a series of linear peptides that inhibit the uPAR/FPR1 interaction and reduce to basal levels directional cell migration, invasion and angiogenesis^37–40^. While providing proof-of-principle for the strategy, none of these peptides represents an ideal lead molecule: many of these peptides are unstable to enzymatic digestion in human serum, which limits their half-life *in vivo*, whereas other peptides exhibit toxicity when administered *in vivo*, probably due to a low affinity binding site to the alpha chain of vitronectin receptor^37–39^. Furthermore, the potent anti-angiogenic peptide Ac-L-Arg-Aib-L-Arg-D-Ca(Me)Phe-NH_2_ (named UPARANT), which is stable in blood and displays prolonged resistance to enzymatic proteolysis, does not inhibit sarcoma cell invasion in a mouse model of lung colonization^40^.

To generate a novel lead series of uPAR/FPR1 inhibitors, we applied the Retro-Inverso (RI) approach^41–44^ to our previously described uPAR/FPR1 inhibitors^38, 40^.

In a RI analog, the sequence of the parent peptide and the chirality of all the amino acids are inverted, yielding a peptide with a high degree of topochemical equivalence to the parent peptide, but with the opposite direction of the backbone amide bonds^41, 42, 44^. When the interaction of the parent peptide with its receptor is dominated by side-chain interactions, the biological activity of the RI analog can be maintained, while the all-D amino acid composition ensures stability to proteases. However, inversion of the amide backbone may result in loss of key main chain hydrogen bonds, and the conformational preferences of the parent and RI peptides may be different^45^. Despite these limitations, several examples of successful application of the RI concept have been reported^46–56^.

The best RI-3 antagonist, which as expected was stable to incubation in serum, inhibited, at nanomolar concentration, migration, matrigel invasion and trans-endothelial migration of human sarcoma cells, and blocked VEGF-triggered endothelial tube formation. Moreover, when administered i. p. once daily for 10 days, it reduced *in vivo* tumor growth, intra-tumoral microvessel density and vascular infiltration by human sarcoma cells in nude mice.

## Results

### Peptide Design

**Figure 1:**
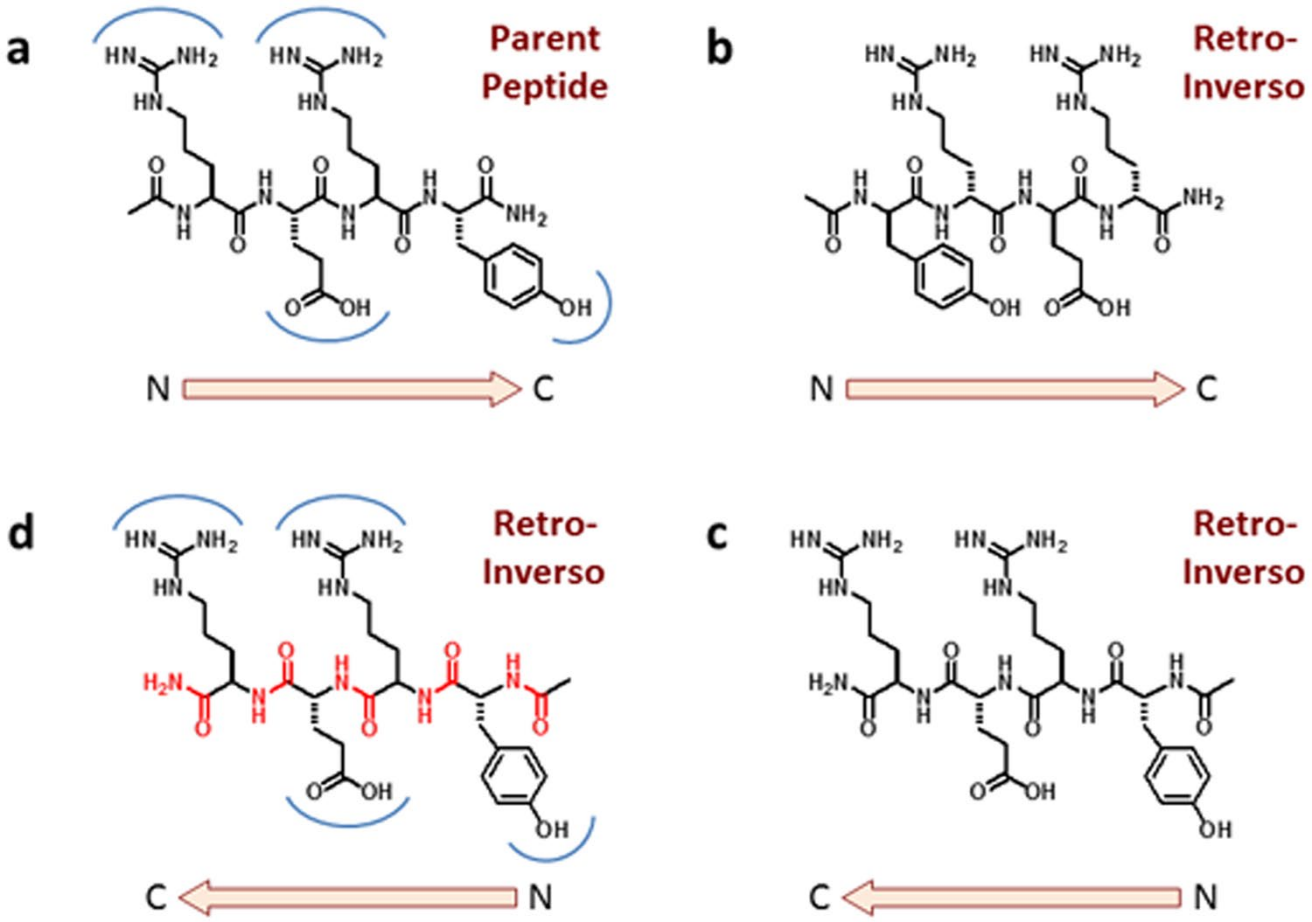
Topological relationship between a peptide and its totally retro-inverso analog, illustrated for Peptide RI-1. (**a**) Parent peptide Ac-Arg-Glu-Arg-Tyr-NH_2_ and (**b**) the corresponding totally Retro-Inverso (RI) analog Ac-(D)-Tyr-(D)-Arg-(D)-Glu-(D)-Arg-NH_2_. The arrow indicates the direction (N-to-C) of the peptide backbone. The blue arcs in (**a**) indicate the binding sites of the side chains. (**c**) The RI peptide in the C-to-N orientation. The topology of the side chains is the same as the parent peptide in the N-to-C orientation. (**d**) In the C-to-N orientation, the side chains of the retro-inverso peptide can occupy the same binding pockets of the parent peptide, while the peptide backbone (red) is inverted, hence the H-bonding pattern is not maintained. The positions of the end-groups are also not equivalent.

One of the limitations of peptides, including those described in our previous studies^37–40^, is susceptibility to degradation by proteases, which can substantially limit their duration of action *in vivo*. One general approach to overcome the problem is the application of the RI concept^41, 43, 44, 57^. In a RI analog, the sequence of the parent peptide is inverted, and the chirality of all the amino acids is changed from L to D. This results in a high degree of topochemical equivalence between the side-chains of the parent peptide and its RI analog, while the backbone amide bonds are inverted (Fig. 1). In a total RI modification, all the amide bonds are reversed, while in a partially modified RI analog only some of the amide bonds are inverted. When the interaction of the parent peptide with its receptor occurs mainly through side-chain interactions, the biological activity of the RI analog should be maintained, while the all-D-amino acid composition ensures stability to proteases. However, inversion of the amide backbone may result in loss of key main chain hydrogen bonds, and the conformational preferences of the parent and RI peptides may be different^45^. Therefore while several examples of biologically active RI peptides have been reported^46–54, 56^, the applicability of the RI concept to a given peptide series must be established case by case.

We designed RI tetrapeptide antagonists of the uPAR/FPR1 interaction (Table 1) using the structure-activity relationship derived from previously described tetrapeptides, in particular Ac-Arg-X1-Arg-X2-NH_2_ (X1 = Glu, Ser, X2 = Tyr, Phe, Trp)^38^, and Ac-Arg-Aib-Arg-Phe-NH_2_^40^.

**Table 1:** Sequence of the RI peptides used in this study.

Peptide	Sequence	MW (theor.)	MH^+^ (found)
RI-1	Ac-(D)-Tyr-(D)-Arg-(D)-Glu-(D)-Arg-NH_2_	663.7	664.4
RI-2	Ac-(D)-Tyr-(D)-Arg-(D)-Ser-(D)-Arg-NH_2_	619.7	620.4
RI-3	Ac-(D)-Tyr-(D)-Arg-Aib-(D)-Arg-NH_2_	621.3	622.3
RI-4	Phenyl-Acetyl-D-Arg-Aib-D-Arg-NH_2_	533.1	533.5
RI-5	3-Phenyl-Propionyl-D-Arg-Aib-D-Arg-NH_2_	547.1	547.5

In addition to the RI tetrapeptides RI-1 – RI-3, we also prepared two RI tripeptides, RI-4 – RI-5, capped with an aromatic carboxylic acid mimicking the side chain of the fourth residue. The purpose of this modification was to maintain the contribution of the side chain to binding, while eliminating a labile amide bond, similar to the strategy outlined by Svenson *et al*.^58, 59^. The sequence of five RI analogs is shown in Table 1.

### The Retro-Inverso peptides inhibit cell migration and matrigel invasion

**Figure 2:**
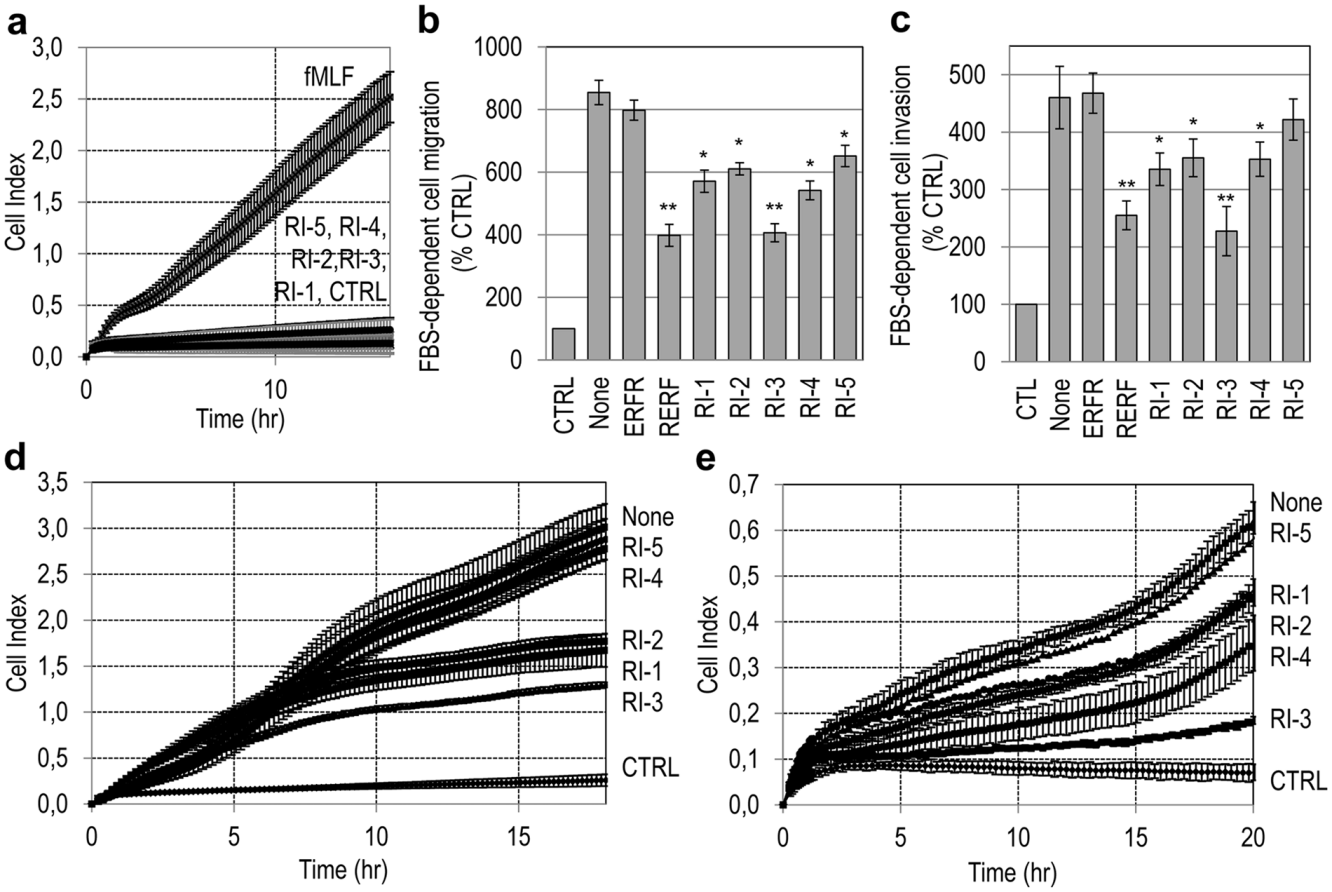
The Retro-Inverso peptides inhibit cell migration and matrigel invasion. (**a**) Human fibrosarcoma HT1080 cell migration toward 10 nM fMLF or 10 nM the indicated peptides, monitored for 18 hr by the xCELLigence system Data represent mean ± SD from a quadruplicate experiment. (**b,c**) HT1080 cells were allowed to migrate (**b**) or invade matrigel (**c**) toward 10% FBS (None) or 10% FBS plus 10 nM of the indicated peptides in Boyden chambers for 4 hr (**b**) or 6 hr (**c**). Basal values assessed in the absence of FBS (CTRL) were taken as 100% and all values were reported relative to that. Data are the means ± SD of three independent experiments, performed in triplicate. *Statistical significance calculated against None, with *P < *0.01*, **P < *0.001*. (**d**,**e**) HT1080 cell migration (**d**) or matrigel invasion (**e**) toward serum-free medium (CTRL), 10% FBS without (None) or with 10 nM of the indicated peptides, monitored for 18 hr (**d**) or 20 hr (**e**) by the xCELLigence system. Data represent mean ± SD from a quadruplicate experiment.

First, we assessed whether the RI peptides elicit cell migration. To this end, we took advantage of the highly mobile and invasive human fibrosarcoma HT1080 cell line, that expresses a considerable amount of uPAR on the cell surface^36, 38^. Cell migration toward 10 nM N-formyl-methionyl-leucyl-phenylalanine peptide (fMLF) or 10 nM the indicated RI peptides was monitored in real-time for 18 hr using the xCELLigence RTCA technology which records as cell index the impedence changes due to the adhesion of migrating cells to microelectrodes. All RI peptides failed to trigger cell migration, unlike fMLF that elicited a considerable cell migration, as expected, (Fig. 2a). We next evaluated the peptides for inhibition of cell migration and invasion of HT1080 cells, in comparison with the previously identified inhibitor RERF^38^. As shown in Fig. 2b, RERF reduced FBS-directed cell migration by 56%, whereas the scramble control peptide ERFR was ineffective, as expected^38^. The peptides RI-1, RI-2, RI-3, RI-4 and RI-5 reduced cell migration by 33%, 29%, 57%, 37% and 28%, respectively.

Cell migration is a prerequisite for cancer cell invasion. Therefore, we investigated whether the RI peptides prevent matrigel invasion of HT1080 cells. In the presence of 10% FBS, HT1080 cells are able to cross matrigel, and RERF can reduce FBS-directed cell invasion by 45% (ERFR is inactive)^38^. As shown in Fig. 2c, RI-1, RI-2, RI-3, RI-4 and RI-5 reduced the extent of cell invasion by 27%, 23%, 51%, 23% and 9%, respectively. Similar results were obtained when HT1080 cell migration (Fig. 2d) and invasion (Fig. 2e) were monitored for 18 or 20 hr, respectively, showing that the inhibitory effect on cell motility and invasiveness persists over time.

### Conformational analysis of RI peptides

**Figure 3:**
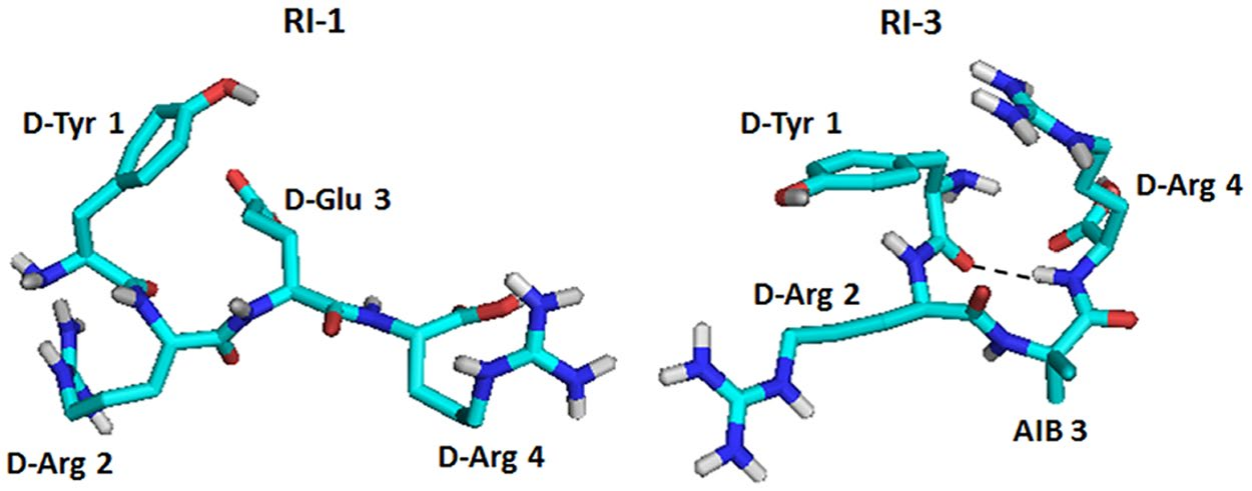
Conformational analysis on Retro-Inverso Peptides. Shown is the average conformation for RI-1 (left) and RI-3 (right) obtained during the MD simulations. In RI-3, the hydrogen bond between the CO group of D-Tyr1 and the NH group of D-Arg4 is shown as a dashed line.

To understand the structural basis of RI-3 inhibitory effect on cell migration and invasion, we decided to run molecular dynamics (MD) simulations on the RI-1, RI-2, and RI-3 tetrapeptides. The peptides were modeled with the Builder module in Insight II, and their conformational fluctuations were analyzed in water at neutral pH, at 300°K. The trajectories during the simulations were analyzed in terms of Root-Mean-Square Deviation (RMSD) and gyration radius (Rg) plots, and secondary structure evolution. The RMSD plots obtained by overlapping the various structures during the simulations suggested that RI-1 and RI-2 are very flexible, whereas RI-3 reached convergence after 20 ns with a mean value of RMSD around 0.3 nm (Supplementary Fig. 1). The Rg plots also indicated that the gyration radii of RI-1 and RI-2 fluctuated more than that of RI-3, where the mean value of Rg decreased from 0.35 to 0.25 nm (Supplementary Fig. 1). Finally, the analysis of the secondary structures confirmed that RI-3 formed a turn structure during the great part of the MD simulation (Supplementary Fig. 2). These data were in agreement with the analysis of the H-bonds conducted on frames after 10, 20, 30, 40, 50, 60, 70, 80, 90 and 100 ns, highlighting for RI-3 the formation of one main chain – main chain (MM) H-bond between the CO group of D-Tyr^1^ and NH group of D-Arg4, specific of turn conformation (Table 2). The structures of RI-1 and RI-3 from the MD simulations are shown in Fig. 3.

**Table 2:** MD simulation on RI-3: H-bonds between the residues.

Frame (ns)	H-bonds
10	CO(D-Tyr1) – NH (Aib3)
20	
30	CO (D-Tyr1) – NH (D-Arg4)
40	
50	CO (D-Tyr1) – NH (D-Arg4)
60	CO (D-Tyr1) – NH (D-Arg4)
70	CO (D-Tyr1) – NH (D-Arg4)
80	CO (D-Tyr1) – NH (D-Arg4)
90	
100	CO (D-Tyr1) – NH (D-Arg4)

The MD results offer an explanation for the antagonist activity of the RI peptides, and in particular for the most active sequence, RI-3. The latter shows a stable turn structure, with the typical β-turn H-bond between the CO of the first residue (D-Tyr1) and the NH of the fourth residue (D-Arg4). This is the same H-bond which was identified by NMR for the antagonist UPARANT^40^, between the CO of Arg1 and the NH of (αMe)Phe4. A turn structure was also found in the antagonist RERF^38^. It appears therefore that despite the inverted amide backbone, the RI peptides can explore the same region of the conformational space as the parent antagonists^38, 40^, and that a more stable turn structure as found in RI-3 yields higher potency. The higher stability of the turn structure in RI-3 is likely due to the presence of the α,α-disubstituted residue Aib in position 2, as seen in UPARANT^40^. Therefore, we focused on RI-3 for a more detailed characterization.

### RI-3 inhibits migration and invasion of HT1080 cells in a dose dependent manner

**Figure 4:**
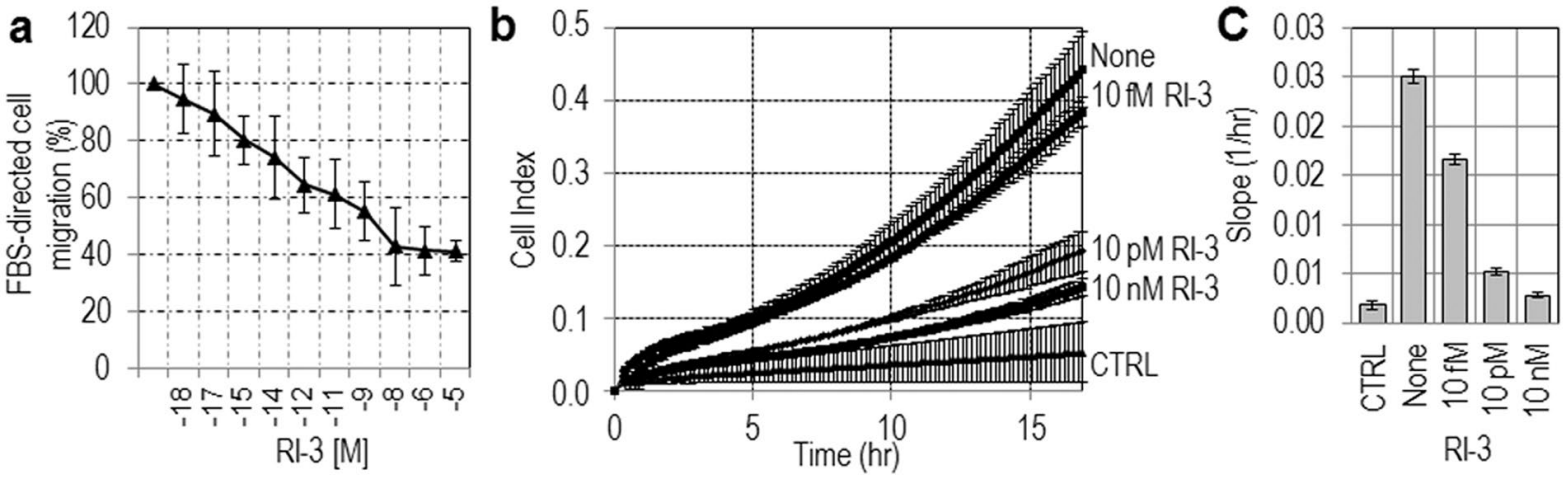
RI-3 inhibits migration and invasion of HT1080 cells in a dose dependent manner. (**a**) HT1080 cells were allowed to migrate in Boyden chambers for 4 hr toward 10% FBS plus increasing concentrations of RI-3. Value assessed in the presence of FBS alone was taken as 100% and all values were reported relative to that. Data are the means ± SD of three independent experiments, performed in triplicate. (**b**,**c**) HT1080 cells were allowed to invade matrigel in real time for 20 hr toward DMEM (CTRL), 10% FBS (None), or 10% FBS plus increasing concentrations of RI-3. Data represent mean ± SD from a quadruplicate experiment. Slopes (**c**) represent the change rate of cell index values generated in a 0–18 hr time frame.

A dose-response curve showed that inhibition of cell migration (Fig. 4a) and cell invasion (Fig. 4b) by RI-3 is dose-dependent. Inhibition of cell migration starts in the high fM range, it seems to level off in the nM range (Fig. 4a). An overall 50% reduction of cell migration (Fig. 4a) and invasion (Fig. 4b,c) was reached at 1 × 10^−13^ M and 1 × 10^−11^ M RI-3, respectively.

### RI-3 inhibits agonist/FPR1 interaction, agonist-induced FPR1 internalization and fMLF-dependent intracellular protein phosphorylation

**Figure 5:**
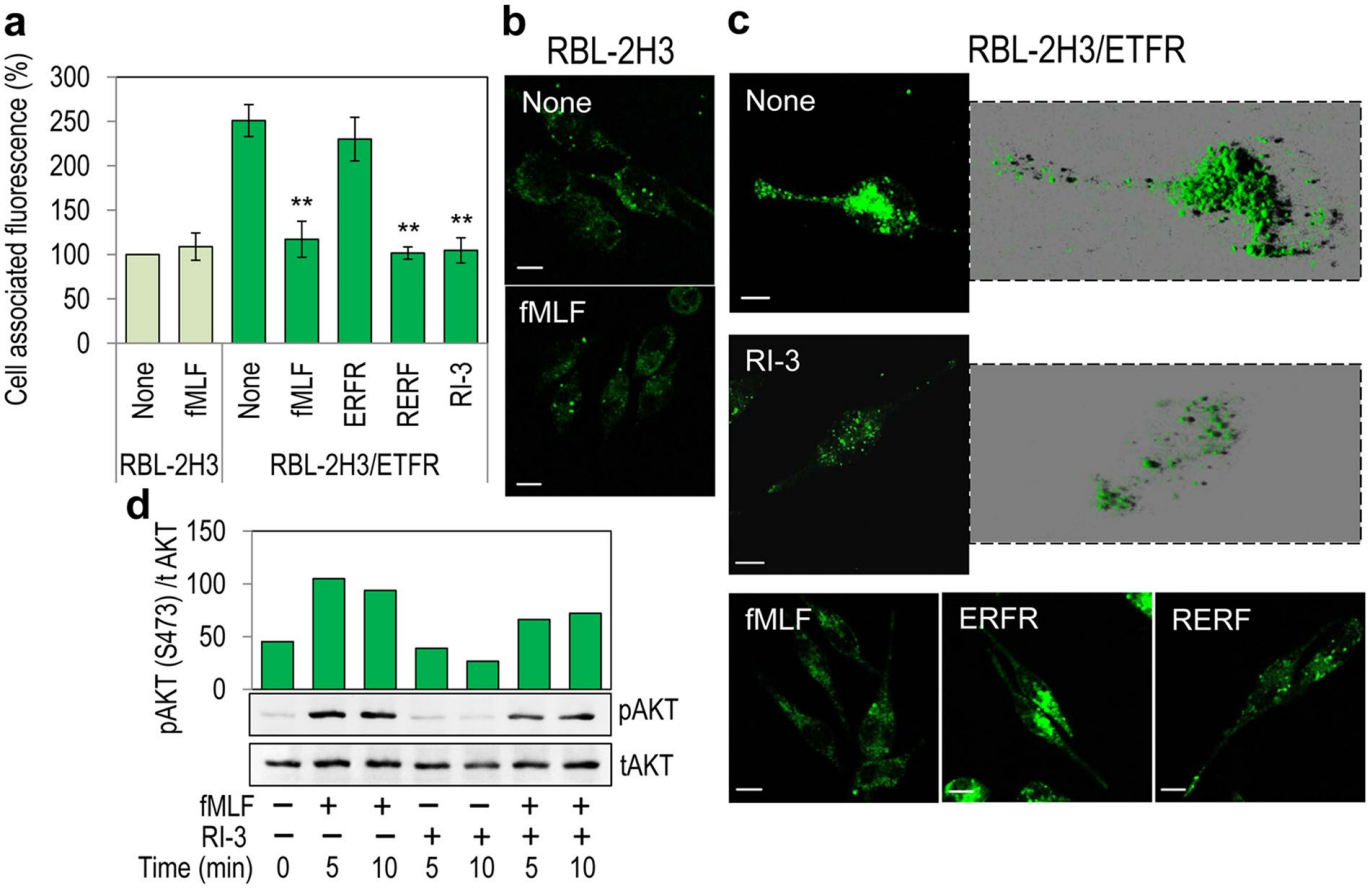
RI-3 blocks agonist/FPR1 interaction, agonist-induced FPR1 internalization and fMLF-dependent AKT phosphorylation. (**a**) RBL-2H3 and RBL-2H3/ETFR cells incubated with diluents (None), 100 nM fMLF, 100 nN RERF, 100 nM ERFR (scrambled peptide control) or 100 nM RI-3 for 60 min at 4 °C, were exposed to 10 nM FITC-fMLF for 60 min at 4 °C. Cell-associated fluorescence was measured using a fluorescence plate reader. Data expressed as a percentage of the fluorescence associated to RBL-2H3 cells exposed to FITC-fMLF alone, and considered as 100%, represent mean ± SD from three experiments performed in triplicate. **Indicates statistical significance against RBL-2H3/ETFR cells exposed to FITC-fMLF alone with *P* = *0.001*. (**b**,**c**) Images of RBL-2H3 (**b**) and RBL-2H3/ETFR (**c**) cells incubated with diluents (None), 100 nM of the indicated peptides for 30 min at 37 °C, exposed to 10 nM FITC-fMLF for 30 min at 37 °C and then visualized using a Zeiss 510 Meta LSM microscope in 2D or 3D projections. Scale bar: 5 µm. Original magnification: 630x. (d) Whole cell lysates from RBL-2H3/ETFR cells (50 µg/sample) immunoblotted with anti-phospho-Akt (pAKT) Ab and then with total anti-Akt (AKT) mAb. The enclosed bar graph shows the average quantification of the pAKT content expressed as a percentage of total AKT. The uncropped images of selected immunoblots are shown in Supplementary Fig. 6a.

RERF inhibits in a dose-dependent manner the directional migration of rat basophilic leukemia RBL-2H3/ETFR cells expressing high levels of FPR1^37^ by preventing the uPAR/FPR1 interaction and, consequently, agonist-triggered FPR1 activation^38^. To test whether RI-3 affects agonist-induced FPR1 activation, rat basophilic leukaemia RBL-2H3 cells devoid of FPR1, and RBL-2H3/ETFR cells stably expressing FPR1, were pre-incubated with diluents (None), an excess of fMLF, RERF, ERFR, or RI-3 for 60 min at 4 °C (to avoid FPR1 internalization), and then exposed to 10 nM N-formyl-Nle-Leu-Phe-Nle-Tyr-Lys-fluorescein (FITC-fMLF) for additional 60 min at 4 °C. RBL-2H3 cells displayed a very low uptake that was not saturable by unlabeled fMLF (Fig. 5a). In contrast, RBL-2H3/ETFR cells exhibited a specific binding that was strongly reduced by unlabelled fMLF and by RERF, but not by ERFR. RI-3 peptide prevented binding of FITC-fMLF to RBL-2H3/ETFR cells to a similar extent as compared to fMLF as well as RERF (Fig. 5a).

To evaluate the effect of RI-3 on agonist-dependent FPR1 internalization, binding experiments were performed at 37 °C. At 37 °C, RBL-2H3 cells failed to uptake FITC-fMLF (Fig. 5b). In contrast, upon exposure of RBL-2H3/ETFR cells to FITC-fMLF (None), FPR1 appeared mainly internalized as indicated by green fluorescent intra-cytoplasmic spots, which were prevented by cell pre-incubation with an excess of fMLF, RERF, but not ERFR. 3D reconstruction of z-stack analysis confirmed the paucity of FPR1 internalization in cells pre-incubated with unlabeled RI-3 as compared with those incubated with diluents (Fig. 5c). To investigate the effects of RI-3 on the phosphorylation of fMLF intracellular targets, like Akt, which is involved in the RERF inhibitory effect^39^, RBL-2H3/ETFR cells were exposed to DMEM or 10 nM fMLF with or without 10 nM RI-3 for 0 to 10 minutes, and phosphorylated proteins were detected by Western blot. As previously observed in endothelial cells exposed to 10 nM RERF^39^, we found that 10 nM RI-3 itself did not affect Akt phosphorylation. Conversely, RI-3 consistently decreased the fMLF- induced Akt phosphorylation (Fig. 5d). To further characterize the inhibition of fMLF-triggered signaling by RI-3, fMLF-dependent phosphorylation of protein kinases in the presence of 10 nM RI-3 was investigated with a dot-blot human phospho-kinase array kit. In the absence of any stimulus, RI-3 did not change phosphorylation of protein kinases. By contrast, cell exposure to fMLF elicited an appreciable increase in the phosphorylation of many proteins (Supplementary Fig. 3a). The addition of 10 nM RI-3 for 10 min caused a reduction in p38α(T180/Y182), ERK1/2(T202/Y204/T185/Y187), Akt(S473), CREB(S133), Fgr(Y412) and FAK(Y397) (21%, 25%, 22%, 42%, 43% and 30%, respectively, Supplementary Fig. 3 a,b). Collectively, these data indicate that RI-3 competes with fMLF for binding to FPR1, and inhibits fMLF-triggered FPR1 internalization. They also suggest that p38 MAPK and PI3K/AKT signaling cascades, which are documented to mediated fMLF-triggered signal transduction pathways^60^, are affected by RI-3.

### RI-3 is stable in human serum

**Figure 6:**
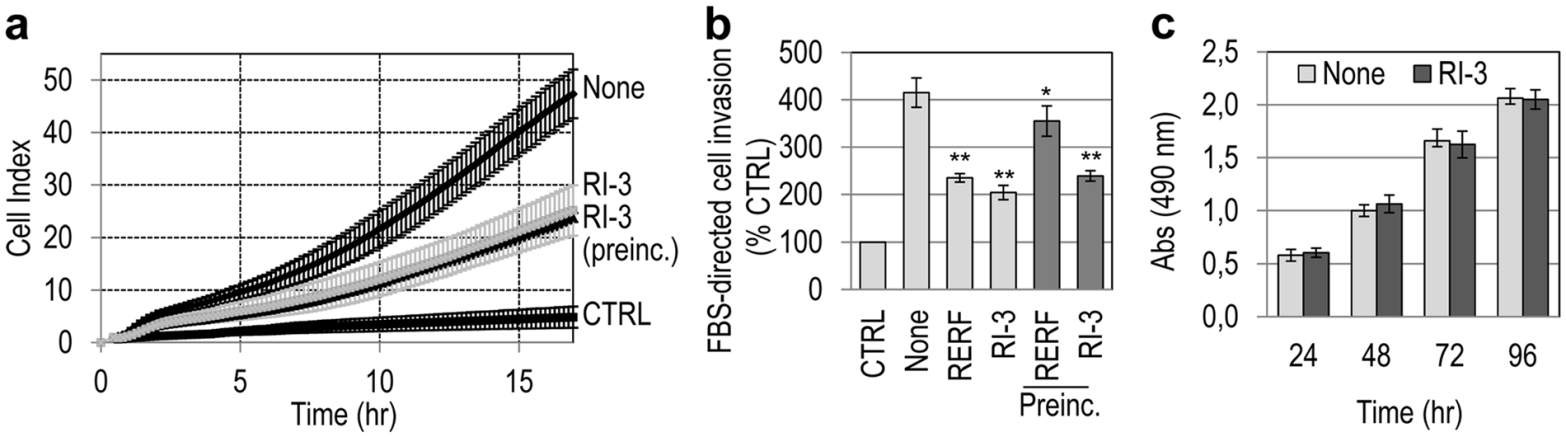
RI-3 is stable in human serum and does not affect cell viability. (**a**) HT1080 cells were allowed to migrate in real time for 18 hr toward DMEM (CTRL), or 10% FBS in the absence (None) or the presence of 10 nM RI-3 or 10 nM RI-3 pre-incubated at 10^−3^ M in bovine serum for 18 hr at 37 °C. Data represent the means from a quadruplicate experiment. (**b**) HT1080 cells were allowed to invade matrigel in Boyden chambers for 6 hr toward DMEM (CTRL), or 10% FBS in the absence (None) or the presence of 10 nM RI-3 or 10 nM RI-3 pre-incubated at a 10^−3^ M in human serum for 18 hr at 37 °C. The basal value assessed in the absence of FBS (CTRL) was taken as 100% and all values were reported relative to that. Data are the means ± SD three independent experiments, performed in triplicate. *Indicates statistical significance calculated against None, with *P < *0.05*, **P < *0.0001*. (**c**) Cell proliferation of HT1080 cells assessed by MTS assay. HT1080 cells were seeded on plates in growth medium plus/minus 10 µM RI-3, and allowed to proliferate at 37 °C in 5% CO_2_. Medium with or without RI-3 was replaced every 24 hr. At the indicated times, the absorbance of adherent cells was assessed with a microplate reader. Data are the means ± SD three independent experiments, performed in triplicate.

A major limitation of peptides for use as therapeutics is susceptibility to degradation by serum proteases. The stability of RI-3 in bovine and human serum was investigated by comparing inhibitory activities of RI-3 and RI-3 pre-incubated at 10^−3^ M with serum for 18 hr at 37 °C on cell migration and invasion. HT1080 cells were allowed to migrate for 18 hr toward DMEM (CTRL), 10% FBS (None), 10% FBS plus 10 nM RI-3 or 10% FBS with 10 nM RI-3 pre-incubated in bovine serum. The Cell index values recorded by migrating cells exposed to RI-3 or RI-3 pre-incubated in bovine serum generated overlapping curves, suggesting that RI-3 is stable to enzymatic digestion (Fig. 6a). The stability of RI-3 in human serum was investigated in cell invasion experiments performed in Boyden chambers. HT1080 cells were allowed to invade matrigel for 6 hr, toward DMEM (CTRL), 10% FBS (None), 10% FBS plus 10 nM RI-3 or 10% FBS with 10 nM RI-3 pre-incubated for 18 hr at 37 °C with human serum. When pre-incubated with human serum, RI-3 retains 81% of inhibitory activity (Fig. 6b).

### RI-3 does not affect cell viability

To rule out that the reduction in the number of migrating or invading cells by RI-3 is due to arrest of cell proliferation and/or cell death, we tested the number and doubling times of cells exposed to growth medium with/without 10 µM RI-3 by a MTS colorimetric method that identifies metabolically active cells (Fig. 6c) and a cell proliferation assay performed using the xCELLigence technology (Supplementary Fig. 4). In both cases, no difference was apparent in the presence or absence of 10 µM RI-3.

### RI-3 prevents matrigel invasion by sarcoma cells from tissues of different origin

**Figure 7:**
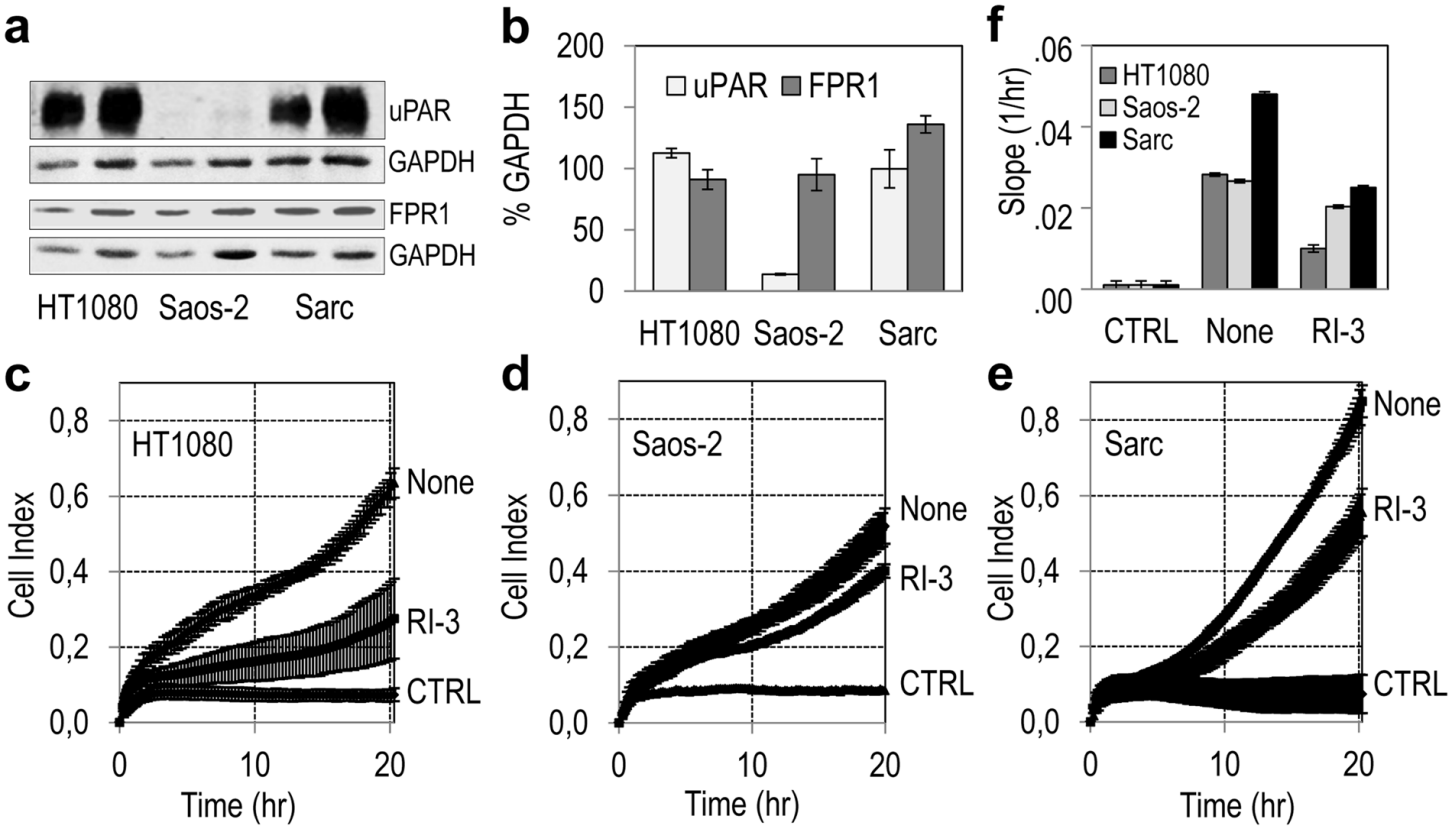
RI-3 prevents matrigel invasion by sarcoma cells from tissues of different origin. (**a**,**b**) Whole cell lysates (20 and 40 μg/sample) from HT1080, Saos-2, and Sarc cells were resolved on a 10% SDS-PAGE, followed by Western blotting with 1 μg/mL R4 anti-uPAR monoclonal antibody or 1 μg/mL anti-FPR1 polyclonal antibody and 0.2 μg/mL anti- GAPDH polyclonal antibody as loading control. The bar graphs show the average quantification of the uPAR/GAPDH and FPR1/GAPDH in three experiments. The uncropped images of selected immunoblots are shown in Supplementary Fig. 6b. (**c**–**e**) Matrigel invasion of HT1080 cells (**c**), Saos-2 (**d**) and Sarc (**e**) cells toward 10% FBS (None) or 10% FBS plus 10 nM RI-3 monitored for 20 hr. Data represent mean ± SD from a quadruplicate experiment. Slopes (**f**) represent the change rate of cell index values generated in a 0–20 hr time frame.

To investigate the ability of RI-3 to prevent matrigel invasion by sarcoma cells derived from human tissues of different origin, human fibrosarcoma HT1080, osteosarcoma Saos-2, and chondrosarcoma Sarc cell allowed to invade matrigel for 20 hr in the absence (None) or in the presence of 10 nM RI-3. As shown by Western blotting analysis of uPAR and FPR1 content in cell lysates, HT1080 and Sarc cells express comparable, high levels of uPAR, whereas very low levels of uPAR were detected in Saos-2 cells. Conversely, FPR1 appeared to be more expressed in Sarc cells than HT1080 and Saos-2 cells (Fig. 7a). These findings were confirmed by densitometric analysis of three independent experiments (Fig. 7b). In the presence of serum, all the cell lines were able to cross matrigel, although to a different extent, and Sarc cells exhibited the highest invasive ability according to their uPAR and FPR1 expression (Fig. 7c–e). RI-3 reduced matrigel invasion of HT1080 and Sarc cells by 64%, and 47%, respectively (Fig. 7f). Interestingly, RI-3 reduced Saos-2 matrigel invasion by 24% only (Fig. 7f), indicating that co-expression of uPAR-FPR is necessary for its inhibitory activity.

### RI-3 inhibits endothelial tube formation, adhesion to the endothelium, and trans-endothelial migration of sarcoma cells

**Figure 8:**
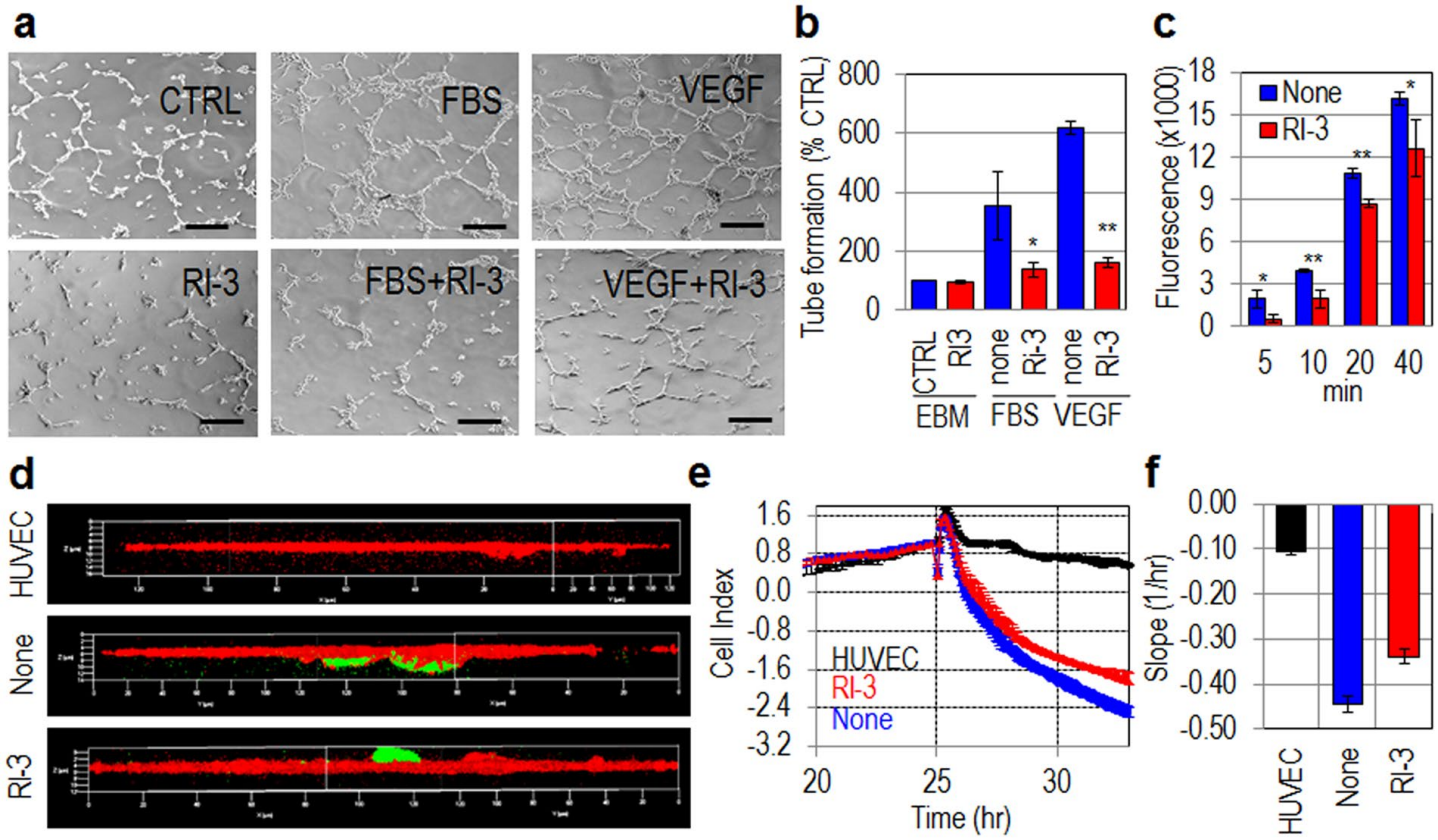
RI-3 prevents *in vitro* endothelial tube formation, adhesion to endothelium and trans-endothelial migration of sarcoma cells. (**a**) HUVECs were suspended in EBM (CTRL) or EBM with 10% FBS or 40 ng/mL VEGF_165_, with/without 10 nM RI-3 and seeded on matrigel-coated plates for 6 hr at 37 °C. Representative pictures were taken with an inverted microscope at 100x magnifications. Scale bar: 100 µm. (**b**) Quantitative analysis of tube formation was calculated as a percentage of tubes formed by cord-like structures exceeding 100 μm in length, counted in the absence of any angiogenic stimulus and considered as 100% (CTRL). Data represent means ± SD of three independent experiments performed in duplicate. Statistical significance was calculated against None with *P < *0.05*, **P < *0.0001*. (**c**) HUVEC were seeded onto matrigel and allowed to attach and to growth for 24 hr prior to seeding GFP-Sarc cells suspended in complete endothelial medium plus/minus diluents (None), or 10 nM RI-3 at 37 °C, 5% CO_2_. At the indicated times, cell associated fluorescence was assessed by a fluorescence plate reader. Data represent means ± SD of three independent experiments performed in duplicate. Statistical significance was calculated against None with *P < *0.05*, **P < *0.0001*. (**d**) After 2 hr, cells were stained with rhodamine-phalloidin and GFP-Sarc cells visualized on multiple z-series collected at 0.20 µm intervals using a confocal microscope. Representative images recorded in 3D projection. Original magnifications: 400x. (**e**) Trans-endothelial migration of Sarc cells. HUVECs (1 × 10^4^ cells/well) suspended in growth medium, were allowed to grow for 25 hr until they form a confluent monolayer, prior to seeding Sarc cells (1 × 10^4^ cells/well) in growth medium plus/minus 10 nM RI-3. Data represent mean ± SD from a quadruplicate experiment. Slopes (**f**) represent the change rate of cell index values generated in a 25–35 hr time frame.

We have previously shown that, upon exposure to uPAR_84–95_, endothelial cells form cord-like structures on matrigel and that peptide inhibitors of the uPAR/FPR1 interaction inhibit angiogenesis *in vitro* and *in vivo* ^27, 39, 40^. We investigated whether RI-3 exerts such effect on morphological differentiation of endothelial cells exposed to pro-angiogenic stimuli. To this end, HUVECs suspended in EBM medium (CTRL), EBM plus 10% FBS or 40 ng/mL VEGF_165_, plus/minus 10 nM RI-3, were seeded on matrigel and the appearance of tubular branches was assessed after 6 hr. Quantitative analysis of tube formation was expressed as a percentage of tubes formed by cord-like structures exceeding 100 μm in length, counted in the presence of EBM alone, considered as 100% (CTRL). As a result, the newly formed cord-like structures assessed in the presence of EBM was 14 +/− 7 tubes per well. In agreement with previous data^39, 40^, both FBS or VEGF_165_ elicited morphological differentiation of endothelial cells into an extensive network of capillary-like structures, consisting of highly organized three-dimensional cords reaching 352% and 620%, respectively, above basal. The addition of 10 nM RI-3 reduced FBS- and VEGF-dependent endothelial capillary-like structures by 61% and 75%, respectively (Fig. 8a,b).

The attachment of tumor cells to the endothelium and their entry into bloodstream are early events occurring during the metastatic process. To ascertain if RI-3 influences tumor cell adhesion to the endothelium, Green Fluorescent Protein (GFP)-tagged Sarc cells were seeded on an endothelial monolayer in the presence/absence of 10 nM RI-3. At the indicated times, non-adherent cells were removed and the cell associated fluorescence was measured using a fluorescence plate reader. GFP-Sarc cells adhere early to endothelium: already after 5–10 min of incubation we found appreciable cell adhesion to endothelium, that increased with time. After 5, 10, 20, and 40 min, 10 nM RI-3 reduced fluorescence by 72%, 50%, 20%, and 22% (Fig. 8c). When the experiment was carried out for 2 hr and co-cultures were labeled for F-actin, the analysis of planes confocal to the endothelium revealed numerous Sarc cells interacting with HUVECs, that decreased in the presence of 10 nM RI-3. Z-stack analysis of the images recorded with 0.20 µm intervals through the entire thickness of the endothelial monolayer and visualized in 3D projection, confirmed that the majority of Sarc cells are confocal to or below the endothelial monolayer in the absence of any treatment, and over the endothelial monolayer in the presence of RI-3 (Fig. 8d). These data indicate that RI-3 prevents the attachment of tumor cells, and suggest that RI-3 may also reduce trans-endothelial migration of tumor cells. The ability of Sarc cells to cross an endothelial monolayer was analyzed using the xCELLigence RTCA technology as previously described^61^. HUVECs were allowed to grow until they formed a monolayer (~25 hr) prior to seeding cells in the presence of EBM-10% FBS plus/minus 10 nM RI-3. At this time, reduction of impedance values, due to invading cells that interrupt monolayer was monitored in real-time for at least 5 hr. An appreciable reduction of endothelial monolayer integrity was achieved by Sarc cells. We found that 10 nM RI-3 reduced the capability of Sarc cells to disrupt endothelial monolayers by 24% (Fig. 8e,f).

### RI-3 exerts anti-metastatic effect *in vivo* on sarcoma cells

**Figure 9:**
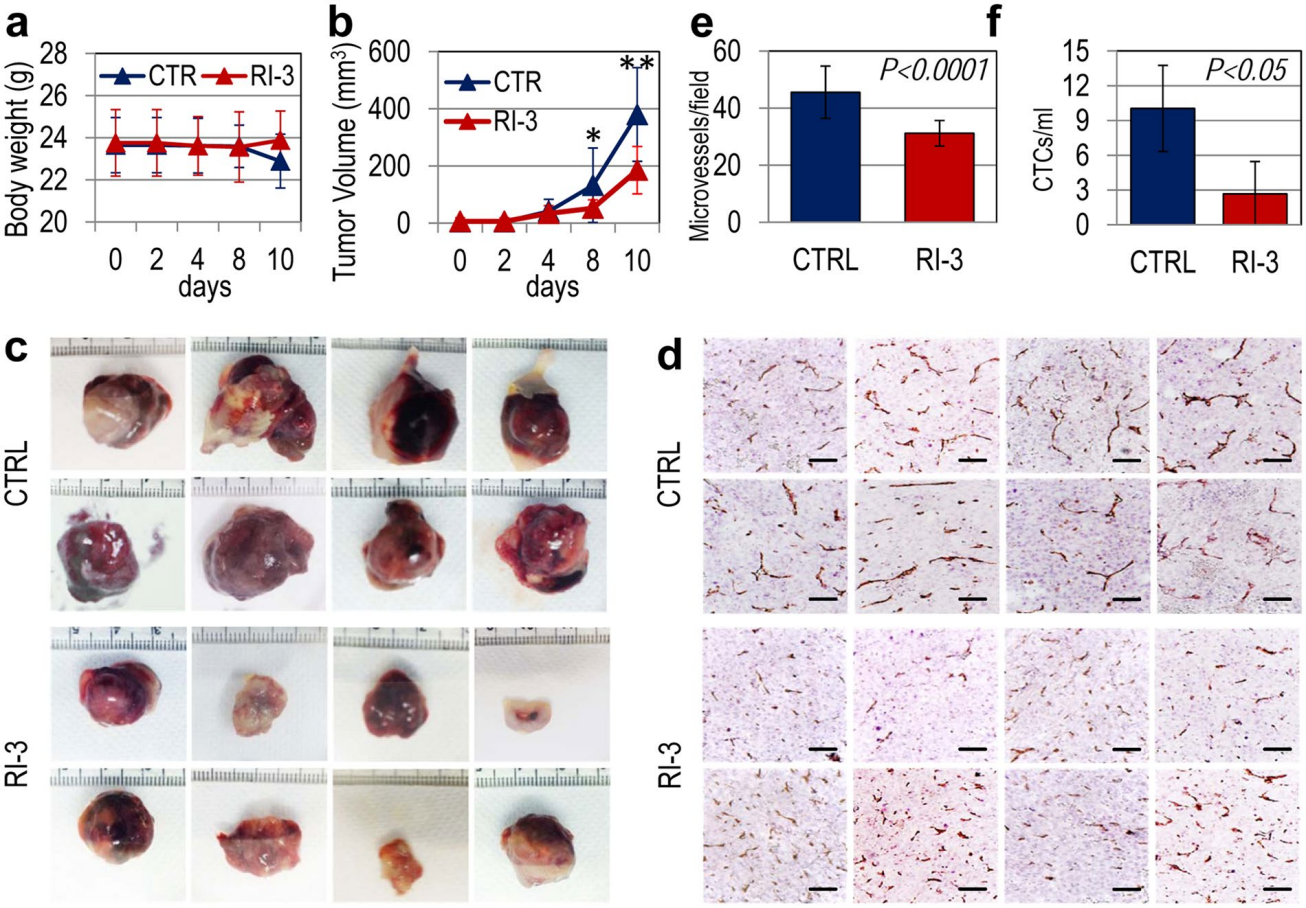
RI-3 reduces tumor growth, intra-tumoral microvessel density and vascular infiltration by sarcoma cells. Sixteen six-eight weeks old, Foxn1nu/nu female nude mice of 23 to 25 g, received an injection of Sarc cells into the right flank as a single-cell suspension (1 × 10^6^ cells in 100 µl of sterile PBS, 96% viability). Eight animals received i.p. injection of 6 mg/kg peptide RI-3 every 24 hr and eight received injections of vehicle only (CTRL). After 10 days, mice were anesthetized and blood samples (~500 µL/mouse) from the retroorbital venous plexus of mice were collected prior to sacrifice by CO_2_ inhalation. (**a**) Body weight measured every 2 days. (**b**) Tumor volumes measured by caliper every 2 days using the formula ½ × (width)^2^ × length (mm), where the width and the length are the shortest and the longest diameters of each tumor, respectively. (**c**) Excised tumors from vehicle-(CTRL) and RI-3*-*treated mice. (**d**) Tumors were fixed in buffered formalin, processed for paraffin sectioning and the sections immuno-stained with CD31. (**e**) Microvessel density was assessed by counting CD31 positive vascular channels in 5 randomly chosen fields per section, in at least two sections per tumor at × 200 magnification. (**f**) DNA from nucleated cells of murine blood samples was extracted and quantitated by Real-Time PCR using primers targeting Alu-sequences. Number of CTCs was calculated by comparing the obtained amplification curves with others generated in spiking experiments which were included in every run.

The above data indicate that RI-3 prevents *in vitro*: invasion of the extracellular matrix, formation of a capillary network, adhesion to the endothelium, and intravasation of sarcoma cells. To assess if these effects also occur *in vivo*, we evaluated inhibitory activity of RI-3 on the growth, vascularization and metastatization of Sarc cells injected in immuno-compromised mice. First, to set up experimental conditions, ten eight weeks-old, Foxn1nu/nu female nude mice received an injection of GFP-tagged Sarc cells having doubling times very similar to the parental cells (Supplementary Fig. 5 a) into the right flank as a single-cell suspension (1 × 10^6^ cells in 100 µl PBS, 96% viability); five animals received i.p-administration of 6 mg/Kg RI-3 every day, while five received injections of vehicle only (CTRL). Tumor growth was monitored daily as brightly visualized by GFP fluorescence with a MacroFluo fluorescence stereomicroscope. Cells readily formed tumors that were visible through the skin as early as 5 days after implantation (Supplementary Fig. 5b). Since signs of tumor ulceration were observed already after 12 days in two mice, all animals were sacrificed. At this time, the visualization of GFP fluorescence, revealed the presence of subcutaneous positive lesions distant at least one centimeter from the primary tumors in 4/5 vehicle-treated and only 2/5 RI-3- treated animals, respectively (Supplementary Fig. 5b, inset), indicating that this tumor has high ability to spread. Next, with the same experimental design, sixteen six-eight weeks-old, Foxn1nu/nu female nude mice received an injection of 1 × 10^6^ Sarc cells into the right flank as a single-cell suspension; eight animals received i.p-administration of 6 mg/Kg RI-3 every day for 10 days, while eight received injections of vehicle only (CTRL). RI-3 was apparently well tolerated, since the weights of mice injected with vehicle or RI-3 were comparable (Fig. 9a). The Sarc cells readily formed tumors, often embedding the surrounding tissues (Fig. 9b,c). The kinetics of tumor formation in vehicle-treated mice was significantly higher than in RI-3-treated mice (Fig. 9b). After 10 days, the volume of tumors from vehicle- and RI-3-treated mice were 330 +/− 163 and 185 +/− 82 mm^3^, respectively (Fig. 9b,c). The difference in tumor size does not depend on a direct inhibitory effect on tumor growth because, *in vitro*, 10 µM RI-3 did not modify cell growth up to 96 hr (Fig. 6c and Supplementary Fig. 4); it likely rather depends on reduction of tumor vascularization, which, in turn, modulates tumor growth. To analyze intra-tumoral vascularization, tumors were fixed in buffered formalin, processed for paraffin sectioning, and vascular channels were counted. We found shorter microvessels (Fig. 9d), and a reduced microvessel density (Fig. 9e) in tumors from RI-3 treated mice *versus* vehicle-treated mice, in line with the ability of RI-3 to prevent *in vitro* formation of a capillary network (Fig. 8a,b). Circulating Tumor cells (CTCs) released into the bloodstream from solid tumors are considered markers of the metastatic process. DNA from nucleated cells of murine blood samples collected just before the sacrifice of vehicle- and RI-3-treated mice, was extracted and quantitated by Real-Time PCR using primers targeting Alu-sequences. Number of CTCs was calculated by comparing the obtained amplification curves with others generated in spiking experiments which were included in every run. We found 10.04 +/− 3.7 CTCs/mL in the blood of 8/8 vehicle-treated mice, and 2.6 +/− 2.7 CTCs/mL in the blood of 4/8 mice treated with RI-3 (Fig. 9f). In the other 4/8 treated mice no CTCs were detected. Although we cannot exclude that the RI-3-dependent decrease in CTC number may also depend on reduction of intra-tumoral vascularization, these findings indicate that RI-3 prevents three key events occurring during the metastatic process of sarcoma cells: extracellular matrix invasion, formation of a capillary network and entry into blood and lymph vessels.

**Figure 10:**
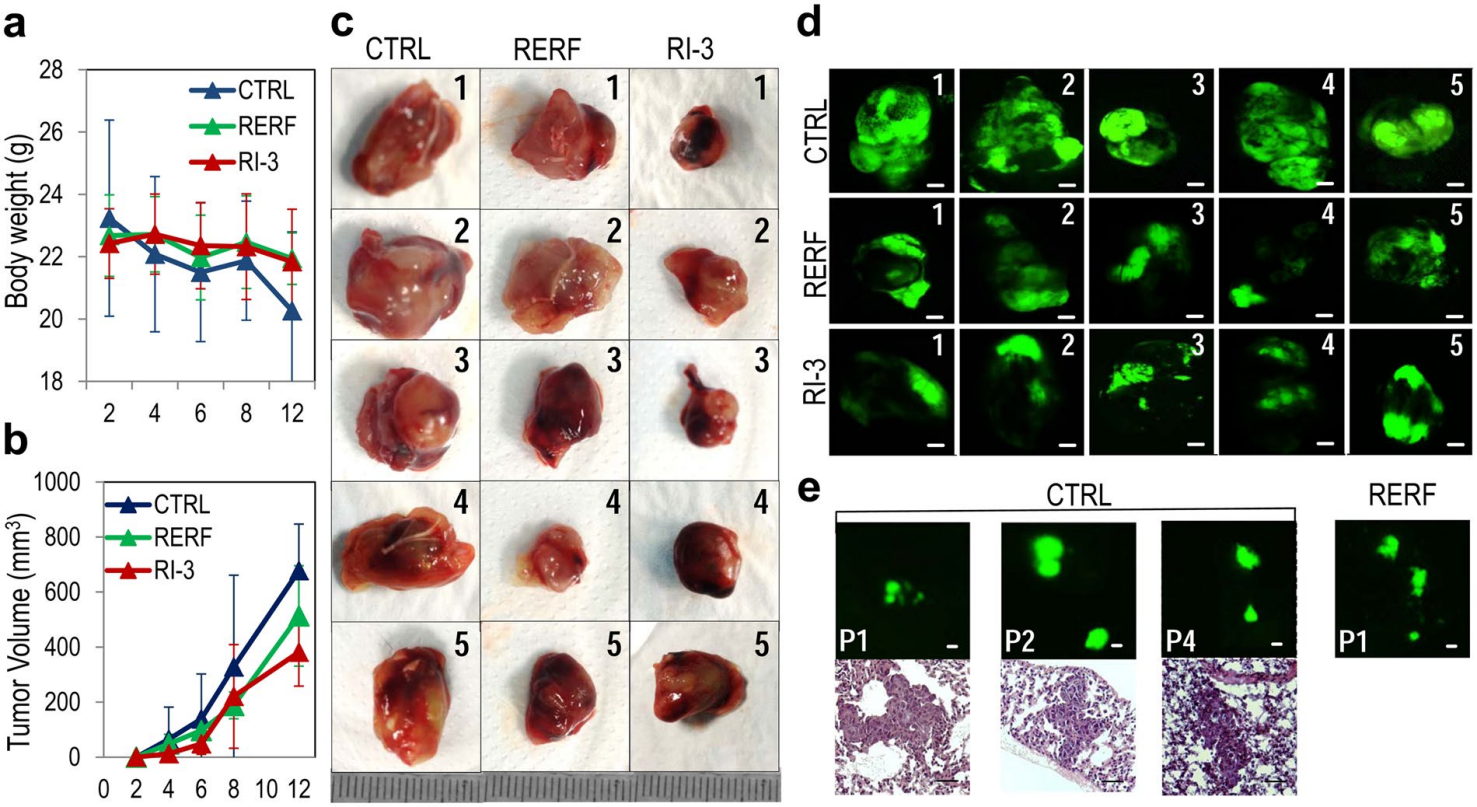
RI-3 exerts anti-metastatic effects *in vivo*. Fifteen six-eight weeks old, Foxn1nu/nu female nude mice of 22 to 25 g, received an injection of GFP-Sarc cells into the right flank as a single-cell suspension (1 × 10^6^ cells in 100 µl of sterile PBS, 96% viability). Ten animals received i.p. injection of 6 mg/kg peptide RI-3 (5 mice) or peptide RERF (5 mice) every 24 hr and five received injections of vehicle only (CTRL). After 12 days, mice were sacrificed (**a**) Body weight measured every 2 days. (**b**) Tumor volumes measured by caliper every 2 days using the formula ½ × (width)^2^ × length (mm). (**c**) Excised tumors from vehicle-(CTRL), RERF- and RI-3-treated mice. (**d**) Fluorescent primary excised tumors visible with MacroFluo fluorescence stereomicroscope. Scale bar: 2 mm. (**e**) Fluorescent positive nodules in excised lungs and H&E stained sections (Scale bar: 50 µm).

To further explore the possibility that RI-3 could prevent the metastatic process of sarcoma cells *in vivo*, fifteen nude mice received an injection of 1 × 10^6^/mouse GFP-tagged Sarc cells into the right flank; ten animals received i.p. administration of 6 mg/Kg RI-3 or 6 mg/Kg RERF (the last as positive control) every day for 12 days (five/group), while five mice received injections of vehicle only (CTRL). RI-3 and RERF treatments did not cause changes in body weight. The kinetics of tumor formation in vehicle-treated mice was faster than in mice treated with RI-3- or RERF (Fig. 10a,b). After 12 days, the volume of tumors from vehicle-, RI-3- and RERF-treated mice were 678 +/− 169, 381 +/− 123, and 512 +/− 183 mm^3^, respectively (Fig. 10b,c). Upon *post hoc* test using Dunnett, the differences in tumor size were significant between the control and RI-3-treated groups (*P* < 0.05), but not between the control and the RERF-treated groups. In keeping with its better stability *in vitro*, RI-3 reduced tumor growth more efficiently than RERF, although the differences between the two treated groups were not significant. MacroFluo analysis of freshly isolated lungs revealed the presence of multiple GFP-positive nodules in 3/5 lungs from untreated mice and 1/5 mouse treated with RERF (Fig. 10e), whereas all lungs from RI-3-trated mice appeared negative. The occurrence of pulmonary micrometastases in CTRL mice was confirmed on serial sections stained with H&E (Fig. 10e). Collectively, our findings indicate that RI-3 reduces tumor growth, tumor vascularization, and prevents formation of lung metastasis from sarcoma cells injected in nude mice.

## Discussion

Despite significant progress in chemotherapy and the spectacular success of cancer drugs with specific molecular targets, overall patient survival has not appreciably improved in the majority of malignancies^62^. This is because the new agents, like the older ones, affect only one characteristic of cancer, uncontrolled growth, while most cancer patients ultimately die from tumor metastases.

Anti-metastasis drugs are particularly attractive since metastasis-associated molecules are generally not mutated in cancer, and are shared among tumor types: for example osteopontin contributes to the invasiveness of over 30 forms of cancer^63^.

However, an understanding of the mechanisms that underlie the metastatic process is accumulating slowly and accordingly, there is a dearth of anti-metastasis drugs^64^. Those currently in clinical development include angiogenesis inhibitors, integrin inhibitors, matrix metalloproteinase inhibitors, chemokine inhibitors, and TGF-β inhibitors^62^.

Since cell migration is central to the metastasis process^1^, and uPAR is central to the regulation of cell migration^5^, drugs which interfere with uPAR signaling may represent novel anti-metastatic agents^12, 65^. However, uPAR signals through multiple complexes^12–19^, and the challenge is to selectively interfere with the more relevant one(s).

We focused our attention of the interaction of uPAR with the formyl-peptide receptor-1 (FPR1), which is involved in progression of solid tumors^32–35^. Aberrant expression of FPR1 has been detected in tumors of different origin, and increased expression of FPR1 in tumors has been reported as a negative prognostic factor^29, 33, 66^. Over the last two decades, several agonists and antagonists of FPR1-dependent functions have been described^67^; however, the majority had off-target effects, discouraging their clinical development.

Gårdsvoll and co-workers proposed a model where uPAR reversibly explores distinct conformational states that translate into different functions. In the absence of uPA, uPAR adopts an “open conformation”, which is unable to induce lamellipodia; uPA engagement or other perturbations of this equilibrium shift the structure to an intermediate, and then to a closed active conformation^22^. Accordingly, stabilization of the closed structure through a DI‒DIII interdomain disulfide bond yields a constitutively active form of uPAR, which bypasses the regulatory role of uPA^22^. In the crystal structure of [C47,C259]uPAR, the linker between the DI–DII domains is more flexible than the boundary between the DII–DIII domains^21, 25^. Ser90 is positioned in a critical “hinge”, which may affect the orientation and position of the DI relative to DII‒DIII domains and as a consequence, uPAR signaling.

We found that the substitution of Ser^90^ with Glu prevented agonist-triggered FPR1 activation and internalization, and potently inhibited uPAR activity^36^. Moreover, short synthetic peptides corresponding to the hinge region could recapitulate the activity of the whole receptor: (i) Ser^88^-Arg-Ser-Arg-Tyr^92^, SRSRY, within the sequence of uPAR_84–95_, showed chemotactic and angiogenic cell activity *in vitro* and *in vivo* ^15–17, 26, 27^, and (ii) introduction of the Ser^90^Glu mutation in the synthetic peptides mimicked the uPAR^S90E^ mutants, yielding antagonists of the FPR1 activation that prevented cell migration and angiogenesis^37–39^.

Peptides have gained increased interest as therapeutics during recent years, and several hundred novel therapeutic peptides are now in preclinical and clinical development^68^. The most common liabilities of this drug class are susceptibility to cleavage by hydrolytic enzymes and rapid renal filtration out of circulation.

In the present work, we sought to overcome the liabilities of our first-generation inhibitors due to their peptidic nature, by applying the retro-inverso (RI) modification. The success of this approach requires that the RI is able to display a side-chain pharmacophore similar to the parent peptide; when this happens, the all-D configuration of the RI ensures stability *in vivo*, unlike the parent L-peptide. MD simulations suggest that this is the case one of the peptides we designed, RI-3, which assumes a turn structure similar to the parent antagonists^38, 40^. Accordingly, we found that RI-3 is the most active RI peptide.

RI-3 maintains, and possibly improves upon, all the features of the parent uPAR/FPR1 antagonists. RI-3 prevents three key steps of the metastatic process: (i) invasion of the extracellular matrix, (ii) entry of tumor cells into blood, and (iii) formation of a capillary network. All three were observed *in vivo* in a sarcoma metastasis model, using RERF as a positive control. RI-3 significantly reduced tumor size, the number of circulating tumor cells, prevented formation of lung metastasis, and reduced the intra-tumoral microvessel density. The latter effect is due to prevention of VEGF-driven angiogenesis. In tumors, an excess of VEGF induces ‘vessel abnormalization’, leading to an increase of interstitial fluid pressure with consequent vessel collapse and impaired drug delivery to tumours^69^.

Interfering with the neovascularization of malignant tumors is one of the most promising therapeutic approaches, one in demand of novel agents, since anti-angiogenic drugs such as bevacizumab display significant side-effects^70^ and the benefits in progression-free survival frequently do not translate into incrased overall survival^71^.

Third, selective impairment of uPAR-mediated FPR1 triggered signaling is expected not to affect other functions regulated by FPR1. We have previously shown that peptide inhibitors of the uPAR/FPR1 interaction prevent phosphorylation of AKT, p38α and ERK1/2, without affecting intracellular calcium mobilization^37, 39^. Accordingly, RI-3 does not elicit any cell response by itself, but prevents *in vitro* the activation of p38 MAPK and PI3K/AKT signaling cascades, which are known to mediate fMLF-triggered signal transduction pathways^60^. Furthermore, RI-3 does not affect cell proliferation *in vitro* and was apparently well tolerated *in vivo* when administered to mice, with no visible side-effects and no change of body weight vs. vehicle-treated animals.

These results for sarcoma are significant, because patients who have metastases at the time of the initial diagnosis are not uncommon, and they have poor prognosis^72^. For these patients, combining current therapy with a systemic anti-metastatic agent might considerably improve the outcome.

Moreover, there is increased awareness that cancer therapy should include, in addition to treatment of the primary tumor and established metastases, also the prevention of metastasis formation^73, 74^. A potent, well-tolerated uPAR/FPR1 antagonist would represent a candidate for such prophylactic therapy.

In summary, we developed novel antagonists of the uPAR/FPR1 interaction based on the retro-inverso concept. The antagonists are stable *in vitro*, and are effective and well tolerated *in vivo*. They represent promising leads to develop an anti-metastasis therapeutic.

## Methods

### Peptide synthesis

The peptides were custom-synthesized on solid-phase with Fmoc/t-Bu chemistry (IRBM Science Park, Pomezia (Rome) Italy). The peptides were purified by reversed-phase HPLC using water/acetonitrile gradients, and characterized by UPLC-MS (Table 1).

### Molecular Modeling and Dynamics Simulations of RI-1, RI-2 and RI-3

Three peptides RI-1, RI-2 and RI-3 were created with the Builder module in Insight II and their conformational fluctuations were analyzed by MD simulations with the GROMACS software and OPLS-AA as force field^75^. Each peptide was embedded in a cubic box filled with SPC216 water molecules (about 1484 for RI-1 and RI-3 and 1489 for RI-2). The peptides were first subjected to energy minimization and position restraints cycles to optimize the system and the location of the water molecules, and then the simulations were carried out with periodic boundary conditions, by adding chloride ions to adjust the net electrostatic charge of the system to zero, as described^76, 77^. All simulations were run for 100 ns at neutral pH and room temperature (300°K). The RMSD, gyration radius fluctuations, number of H-bonds, and secondary structure evolution were evaluated by the GROMACS routine and the Hbplus program^78^.

### Cell lines

Human fibrosarcoma HT1080 and human osteosarcoma Saos-2 were purchased from ATCC, rat basophilic leukaemia RBL-2H3 and RBL-2H3/ETFR cells^37^ were kindly provided by F. Blasi (IFOM, Milan, Italy), and chondrosarcoma Sarc cells derive from a human chondrosarcoma primary culture^79^. All cell lines were grown in Dulbecco Modified Eagle Medium (DMEM) supplemented with 10% fetal bovine serum (FBS), 100 IU/mL penicillin and 50 μg/mL streptomycin. Sarc transfected cells, stably expressing Green Fluorescent Protein (GFP), were obtained using pEGFP-N1 vector (Clontech) and polyfectamine transfection reagent (Quiagen). G418-resistant cells expressing the highest levels of GFP were isolated and amplified. Human umbilical vein endothelial cells (HUVEC)s (Lonza) were grown in Eagle Basal Medium (EBM) supplemented with 4% FBS, 0.1% gentamicin, 1 µg/mL hydrocortisone, 10 µg/mL epidermal growth factor and 12 µg/mL bovine brain extract (Cambrex, Bio Science), and were employed between the third and the seventh passage.

### Cell migration and invasion in Boyden chambers

Cell migration in Boyden chambers was carried out for 4 hr at 37 °C in humidified air with 5% CO_2_, as described^40^. Briefly, cell suspension (1 × 10^5^ viable cells/mL serum free medium) was seeded in each upper chamber. Lower chambers were filled with DMEM alone, DMEM containing RI peptides, 10 nM fMLF, or 10% FBS with/without RI peptides. The two compartments were separated by 8 μm pore size polycarbonate filters (Neuroprobe) coated with 2.5 µg/mL vitronectin (Corning). For the invasion assays, 8 μm pore size filters were coated with 50 μg/filter matrigel (BD Biosciences) as previously described^40^ and cells (3 × 10^4^ viable cells/well) were allowed to invade matrigel for 6 hr at 37 °C, 5% CO_2_. In all cases, at the end of the assay, cells on the lower filter surface were fixed with ethanol, stained with haematoxylin and 10 random fields/filter were counted at 200× magnification. Each experiment was performed three times in triplicate.

### Migration kinetic of cells monitored in real time

The kinetics of cell migration was monitored in real time using the xCELLigence Real Time Cell Analysis (RTCA) technology (Acea Bioscience) as described^61, 80^. For these experiments we used CIM-plates which are provided with interdigitated gold microelectrodes on the bottom side of a filter membrane interposed between a lower and an upper compartment. The lower chamber was filled with serum-free medium or growth medium with/without RI peptides. Cells (2 × 10^4^ cells/well) were seeded on filters in serum-free medium. Cell migration was monitored for 18 hr, and each experiment was performed at least twice in quadruplicate.

### Kinetics of cells invasion

This assay was performed using E-well plates and the xCELLigence RTCA technology as described^61^. Bottom wells were coated with 20 µg/well matrigel diluted in serum free medium. Matrigel was allowed to polymerize for 1 hr at 37 °C prior to seeding cells (1 × 10^4^ cells/well) suspended in growth medium plus/minus RI peptides. Cells that cross matrigel adhere to the bottom of plates causing impedance changes that are proportional to the number of invading cells, and are expressed as a cell index value. Matrigel invasion was monitored for 20 hr. The experiments were performed three times in quadruplicate.

### Ligand binding assay

RBL-2H3 cells, RBL-2H3/ETFR cells stably expressing FPR1 (1.5 × 10^6^ cells/sample) were pre-incubated with diluents or the indicated unlabeled peptides for 60 min at 4 °C, extensively rinsed with phosphate buffer saline (PBS), exposed to 10 nM FITC-fMLF (Molecular Probes) in PBS for additional 60 min at 4 °C and again rinsed with PBS. Cell-associated fluorescence was quantitated with the fluorescence plate reader Victor 3 (Perkin Elmer) using 485 nm excitation and 535 nm emission filters. The experiments were performed three times in triplicate.

### Fluorescence microscopy

Cells grown on glass slides to semi-confluence were exposed to diluents or the indicated unlabeled peptides for 30 min at 37 °C, then to 10 nM FITC-fMLF in PBS for additional 30 min at 37 °C and extensively rinsed with PBS. To visualize the cytoskeleton, the cells were fixed with 2.5% formaldehyde, permeabilized with 0.1% Triton X-100 for 10 min at 4 °C, and incubated with 0.1 µg/mL rhodamine-conjugated phalloidin (Sigma-Aldrich) for 40 min. In all cases, coverslips were mounted using 20% (w/v) Mowiol, visualized with a Zeiss 510META-LSM microscope (Carl Zeiss), and z-series were collected at 0.20 µm intervals.

### Western blotting

Cells detached using 200 mg/L EDTA, 500 mg/L trypsin (Cambrex), were lysed in RIPA buffer (10 mM Tris pH 7.5, 140 mM NaCl, 0.1% SDS, 1% Triton X-100, 0.5% NP40) containing protease inhibitor mixture. Protein content of cell lysates was measured by a colorimetric assay (BioRad). Twenty and forty µicrograms of proteins from each cell lysate were separated on 10% SDS-PAGE and transferred onto a polyvinylidene fluoride membrane. The membranes were blocked with 5% non-fat dry milk and probed with 1 μg/mL R4 anti-uPAR monoclonal antibody recognizing uPAR D3 domain, 1 μg/mL anti-FPR1 polyclonal antibody (Abcam), or 0.2 μg/mL GAPDH Ab (Santa Cruz Biotechnology). Washed filters were incubated with horseradish peroxidase-conjugated anti-mouse or anti-rabbit antibody and detected by ECL (Amersham-GE Healthcare). Densitometry was performed using the NIH Image 1.62 software (Bethesda, MD). Each experiment was performed three times.

### Phospho-kinase array

RBL-2H3/ETFR grown to semi-confluence, were exposed to DMEM alone, 10 nM fMLF with/without 10 nM RI-3 at 37 °C in humidified air with 5% CO_2._ At the indicated times, cells were lysed in RIPA buffer and cell lysate protein content was measured by a colorimetric assay. Western blot analysis of AKT phosphorylation was performed as previously described using anti-phospho-Akt (Ser473) (pAKT) Ab (Cell Signaling) and anti-Akt mAb (R&D System). The effects of fMLF with/without 10 nM RI-3 were analyzed at the level of global kinase phosphorylation, using the dot-blot Proteome Profiler^TM^ Array Human PhosphoKinase Array Kit (R&D Systems), according to manufacturer’s instructions. Briefly, 300 µg of protein were applied on each membrane and the signals were detected as described for Western blot. The pixel density of each spot was measured using NIH Image 1.62 software. The intensity of positive control spots was used to normalize results between the four membranes. The intensity for each spot was then averaged over the duplicate spots.

### Cell proliferation

Cell proliferation was assessed using the xCELLigence RTCA technology as described^61^. Cells (2 × 10^3^/well) were seeded in E-well plates in growth medium and left to growth for 85 hr in the presence or the absence of 10 µM RI-3. For MTS assay, cells seeded on plates in growth medium plus/minus 10 µM RI-3, were allowed to proliferate at 37 °C in 5% CO_2_ and the absorbance of adherent cells stained with tetrazolium/formazan was assessed with a microplate reader. In both cases, medium with or without RI-3 was replaced every 24 hr. Data are the means ± SD of three independent experiments, performed in triplicate.

### Endothelial Cell Tube Formation Assay

The formation of vascular-like structures was assessed on a matrigel basement membrane matrix preparation. HUVEC (4 × 10^4^ cells/well) were suspended in 300 µl pre-warmed serum-free medium. Diluents or effectors were added to the cell suspension prior to seeding cells on polymerized growth factor-reduced matrigel (100 µl/well) (Becton Dickinson). Assays were carried out for 6 hr at 37 °C in humidified air with 5% CO_2_ as described^27^. To quantify tube formation, five random areas/well at 100× magnification were imaged and the number of tubes formed by cord-like structures exceeding 100 μm in length, measured using Axiovision 4.4 software (Carl Zeiss), were counted^27^. The experiments were performed three times in duplicate.

### Cell adhesion onto endothelium

GFP-tagged Sarc cells were seeded on an endothelial monolayer as previously described^28^. Briefly, sterile round glass coverslips (12 mm in diameter) were coated with 1:8 diluted matrigel (Becton Dickinson). HUVEC (5 × 10^4^ cells in 200 μL/well) were seeded onto matrigel and allowed to form a monolayer for ~24 hr at 37 °C, 5% CO_2_ prior to seeding GFP-Sarc cells (1 × 10^4^ cells/well) suspended in complete endothelial medium plus diluents or 10 nM RI-3. At the indicated times, cell-associated fluorescence was assessed by reading cells with the fluorescence plate reader. In a subset of experiments, after 2 hr, cells were stained with rhodamine-conjugated phalloidin and green fluorescent Sarc cells were identified on multiple z-series collected at 0.20 µm intervals using a confocal microscope (Carl Zeiss).

### Trans-endothelial migration

Trans-endothelial migration assays were performed using the xCELLigence RTCA technology as described^61^. Briefly, HUVECs (1 × 10^4^ cells/well) suspended in growth medium, were seeded in E-plates and allowed to grow for ~25 hr, until they form a confluent monolayer, prior to seeding Sarc cells (1 × 10^4^ cells/well) in growth medium plus/minus 10 nM RI-3. When HUVECs are challenged with crossing cells, there is a drop in electrical resistance that is monitored in real-time as cell index changes due to the rupture of the endothelial monolayer. The experiment was performed twice in quadruplicate.

### Growth and vascularization of tumors in mice

To evaluate the effect of RI-3 on tumor growth and vascularization, Sarc cells were injected, as a single-cell suspension (1 × 10^6^ cells in 100 µl of sterile PBS, 97% viability), subcutaneously in the flanks of sixteen six-eight weeks old, Foxn1nu/nu female nude mice (Harlan). Animals were randomized into two 8-mice groups with the treatment group receiving 6 mg/kg RI-3 by intra-peritoneal injection every 24 hr, and the control group receiving an equivalent injected volume of vehicle (PBS) only. Time-dependent average weight was monitored every two days. The length and the width of tumors were measured at different time points with a calliper and volumes were calculated using the formula: ½ × (width)^2^ × length (mm). After 10 days, blood samples (at least 500 µL/mouse) from the retro-orbital venous plexus of mice anesthetized with 1% isoflurane were collected using a heparinized capillary tube and processed for determination of the Circulating Tumor Cells (CTCs). Then, the animals were sacrificed, the excised tumors were fixed in buffered formalin and processed for paraffin sectioning. Tumor vascularization was assessed by counting vascular channels harbouring red blood cells on CD31 immuno-stained sections in 5 randomly chosen fields per section, in at least two sections per tumor at x 200 as described^61^. Two additional experiments were carried out with the same experimental design by injecting GFP-tagged Sarc cells (1 × 10^6^ cells/mouse) subcutaneously in the flanks of twenty-five six-eight weeks old, Foxn1nu/nu female nude mice (Harlan). Animals were treated with 6 mg/kg RI-3, 6 mg/kg RERF or vehicle by intra-peritoneal injection every 24 hr for 10 or 12 days. Tumor growth and the appearance of positive tumor lesions near tumors and in lungs were monitored as brightly visualized by GFP fluorescence with a MacroFluo fluorescence stereomicroscope (Leica). We searched for the presence of GFP-positive nodules on the entire surface of freshly isolated lungs. Lungs were also sliced at approximately 3-mm thickness to examine the parenchyma in depth, and the positive nodules were acquired at higher magnification.

### Isolation and enumeration of Circulating Tumor Cells

To quantify CTCs, DNA from nucleated cells of murine blood samples (500 μl/mouse) was extracted using the QIAamp DNA Mini Kit (Qiagen), according to the manufacturer’s protocol. Quantitative RT-PCR (7900 HT Fast Real-Time PCR System, Applied Biosystems) was performed using 18 ng DNA and the SYBR Select Master Mix (Applied Biosystems) as described. Primers to human Alu-sequences [FW 5′-CACCTGTAATCCCAGCACTTT-3′/RV 5′-CCCAGGCTGGAGTGCAGT-3′] were employed to a final concentration of 0.5 μM. The number of CTCs was calculated by comparing the obtained Ct with a standard amplification curve generated in spiking experiments (1 to 50 cells were collected by pipetting under microscopic control) which were included in every run. DNA from murine blood was included as a negative control.

### Statistical Analysis

The results are expressed as the means ± standard deviations of the number of the indicated determinations. Data were analyzed by one-way ANOVA and *post hoc* Dunnett t-test for multiple comparisons. P < 0.05 was accepted as significant.

### Ethics statement

All experimental protocols were performed in accordance with guidelines of the Istituto Nazionale Tumori “Fondazione G. Pascale”-IRCCS (Quality System n. LRC 6019486/QMS/U/IT- 2015 certificated in conformity with UNI EN ISO 9001:2008). The care and use of animals was approved by Institutional Ethical Committee of Istituto Nazionale Tumori “Fondazione G. Pascale”-IRCCS, Naples, Italy and by the Italian Ministry of Health (protocol n.1185/2016-PR).

## Electronic supplementary material

Supplementary Information
