# Supplementary material for: Retro-inverso Urokinase Receptor Antagonists for the Treatment of Metastatic Sarcomas
Source: Sci Rep. 2017 May 2;7:1312. doi: 10.1038/s41598-017-01425-9 (PMC5430962; doi:10.1038/s41598-017-01425-9)

Supplementary Information

Retro-inverso Urokinase Receptor Antagonists for the Treatment of Metastatic Sarcomas

Maria Vincenza Carriero[1*], Katia, Bifulco[1], Vincenzo Ingangi[1], Susan Costantini[1], Giovanni Botti[1], Concetta Ragone[1], Michele Minopoli[1], Maria Letizia Motti[1], Domenica Rea[1], Giosuè Scognamiglio[1], Gerardo Botti[1], Claudio Arra[1], Gennaro Ciliberto[1], and Antonello Pessi[2*].

[1]IRCCS Istituto Nazionale Tumori "Fondazione G. Pascale" - Naples, Italy.
[2]Peptipharma, Viale Città D'Europa 679, 00144 - Rome, Italy.

*Corresponding Authors:
Maria Vincenza Carriero, m.carriero@istitutotumori.na.it
Antonello Pessi, a.pessi@peptipharma.it

Supplementary Figure 1. RMSD and gyration radius (Rg) plot for RI-1 (a and d), RI-2 (b and e) and RI-3 (c and f) during MD simulations

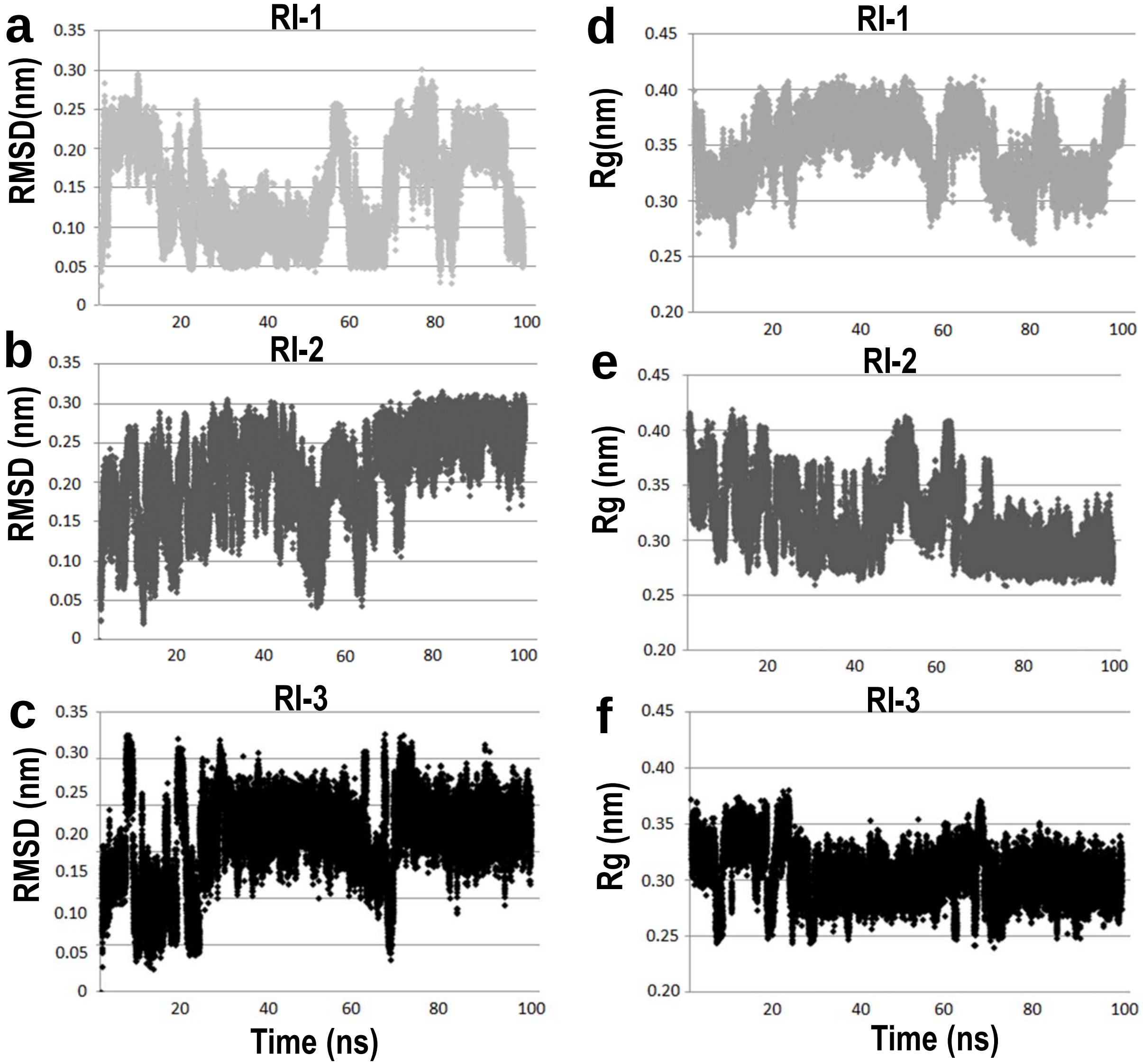

Supplementary Figure 2: Secondary structure evolution for RI-3 peptide during MD simulation. Coil and turn structures are shown as white and yellow lines, respectively

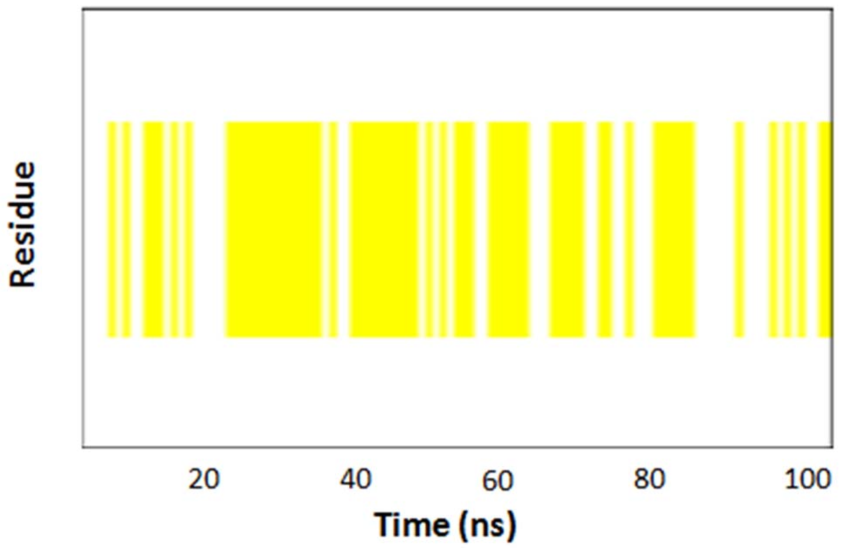

Supplementary Figure 3: fMLF-dependent intracellular protein phosphorylation

a

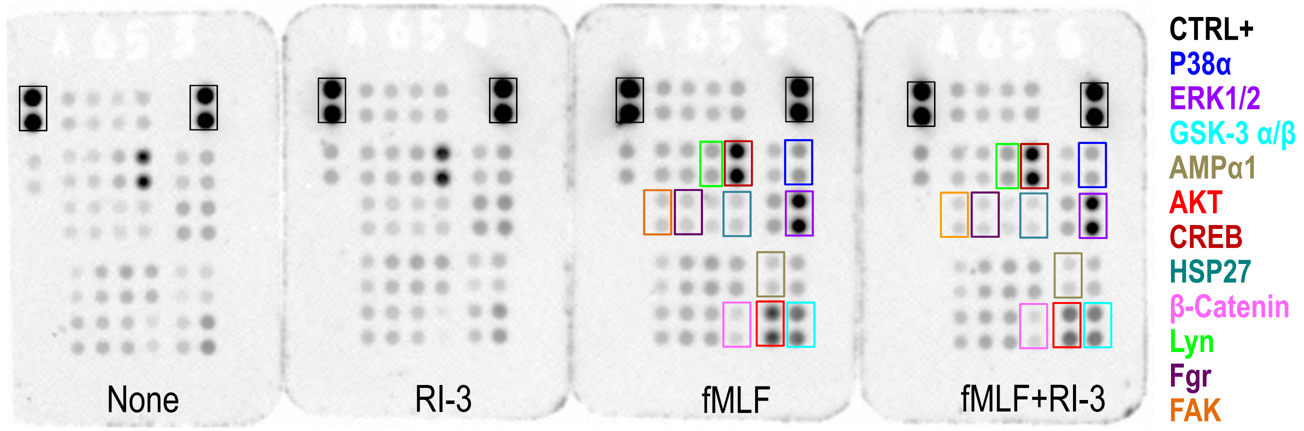

b

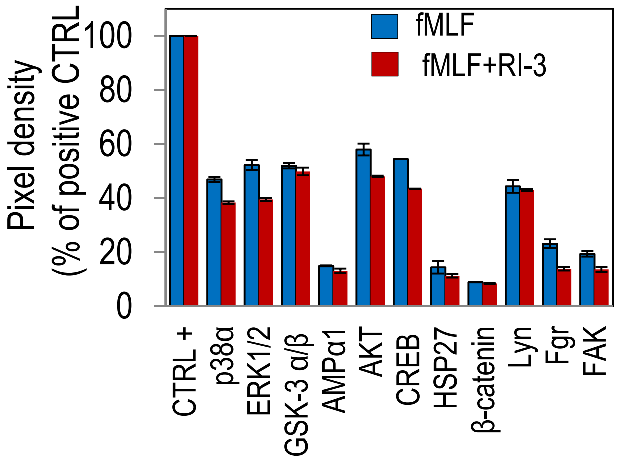

(a) Analysis of RBL-2H3/ETFR cell lysates (200 μg/sample) by a phospho-kinase array (uncropped images). Stained squares indicate the relevant results. (b) Quantification of phospho-kinase array. The pixel density of each spot was measured using NIH Image 1.62 software. The intensity of positive control spots (CTRL+) was used to normalize results between the three membranes. Data expressed as percentage of each positive control were averaged over the duplicate spots.

Supplementary Figure 4: Cell proliferation of HT1080 cells monitored for 75 hr using the xCELLigence technology

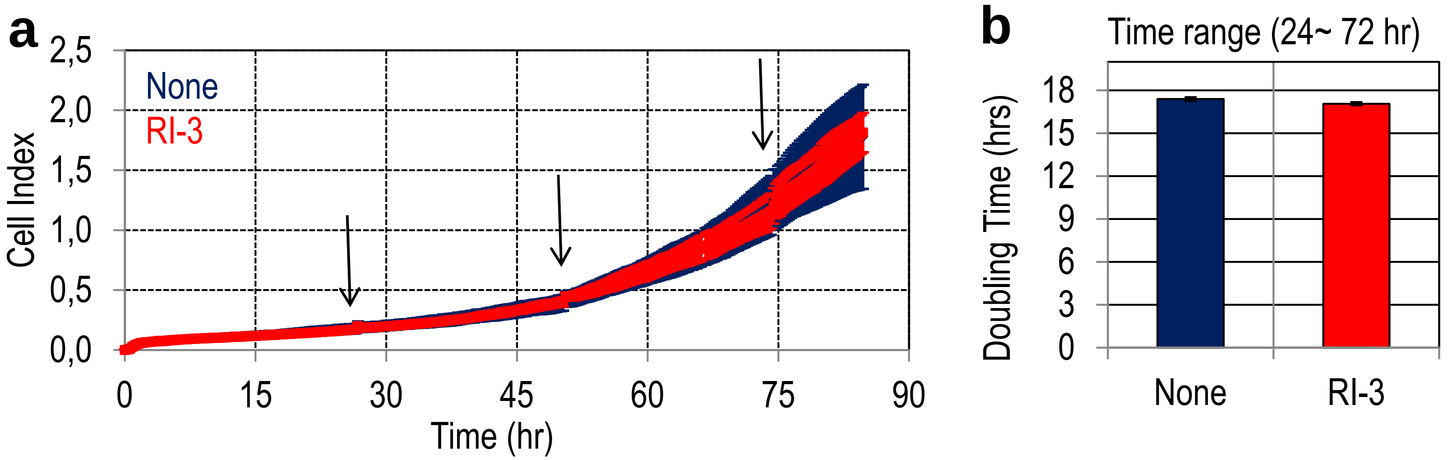

(a) Cells (1x10[3]cells/well) were seeded on plates in growth medium plus/minus 10 µM RI-3 and allowed to proliferate at 37°C, 5% CO2. Medium was replaced every 24 hr (arrows). Impedance value of each well was automatically monitored and expressed as Cell Index. (b) Doubling times calculated from the cell growth curves, in the 12-72 hr time range. Data represent mean ± SD from a quadruplicate experiment.

Supplementary Figure 5. Doubling index of GFP-Sarc cells implanted in nude mice

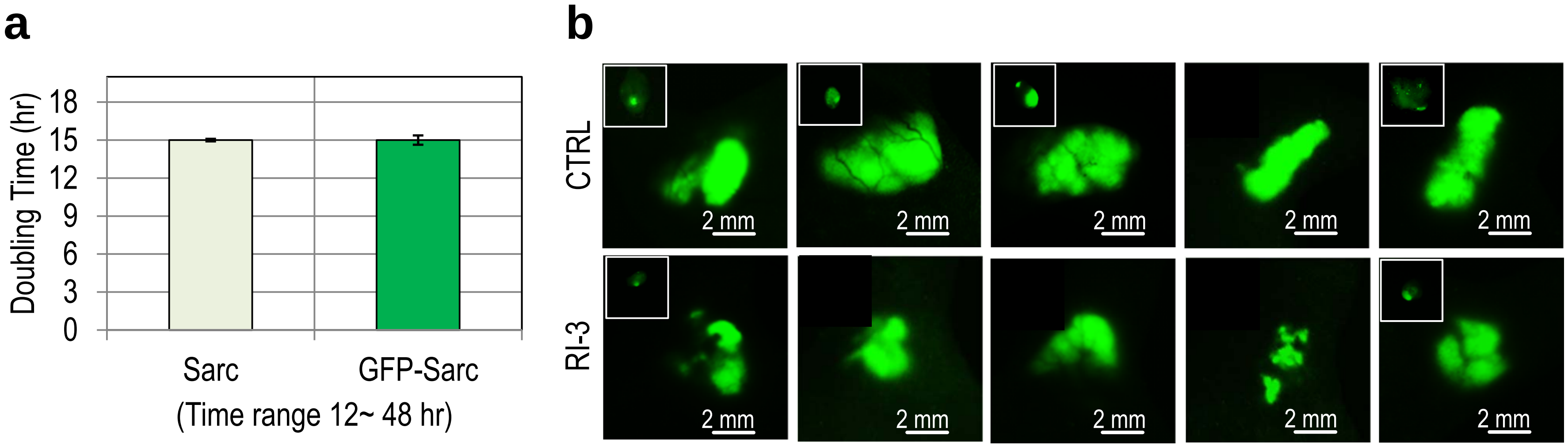

(a) Doubling times calculated from the cell growth curves of Sarc and GFP-Sarc cells, during exponential growth. Data represent mean ± SD from a quadruplicate experiment. (b) Fluorescent primary tumors visible with MacroFluo fluorescence stereomicroscope through the skin 5 days after implantation of human GFP-Sarc cells injected into the right flank of ten Foxn1nu/nu female nude mice. Insets: positive lesions distant at least one centimeter from the primary tumors visualized after 10 days.

Supplementary Figure 6. Uncropped images of immunoblots from Fig.5d (a) and Fig.7a (b)

a

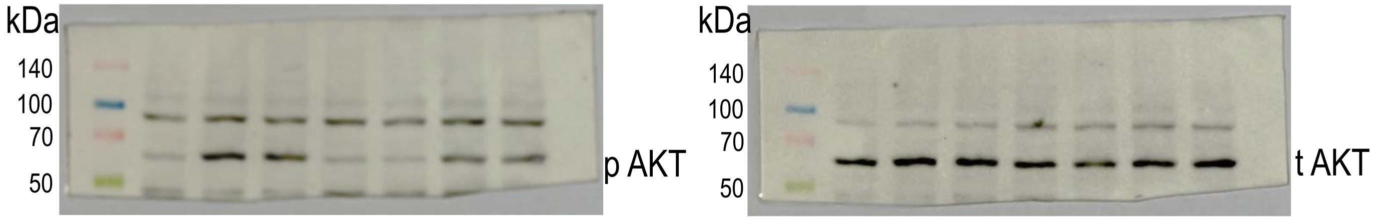

b

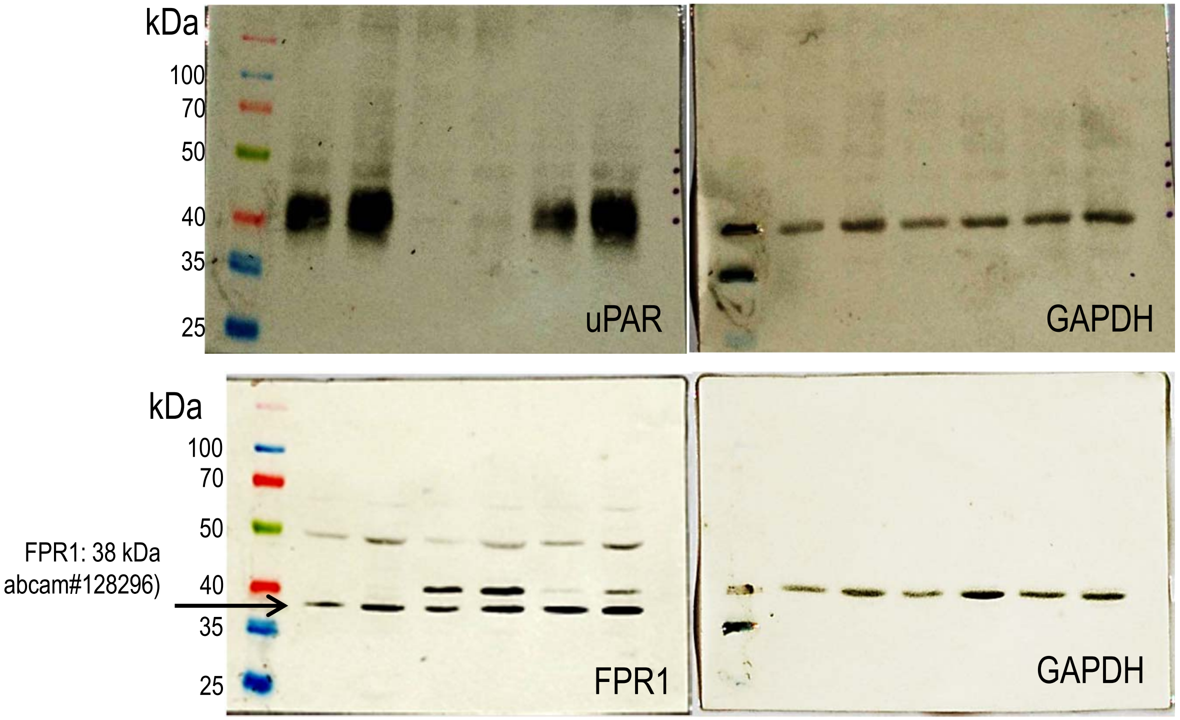

Supplement: Supplementary file 1 — Supplementary Information [file 41598_2017_1425_MOESM1_ESM.pdf]
